# Thermal Habitat Index of Many Northwest Atlantic Temperate Species Stays Neutral under Warming Projected for 2030 but Changes Radically by 2060

**DOI:** 10.1371/journal.pone.0090662

**Published:** 2014-03-05

**Authors:** Nancy L. Shackell, Daniel Ricard, Christine Stortini

**Affiliations:** 1 Oceans and Ecosystem Science Division, Department of Fisheries and Oceans, Bedford Institute of Oceanography, Dartmouth, Nova Scotia, Canada; 2 Biology Centre AS CR v.v.i., Institute of Hydrobiology, České Budějovice, Czech Republic; 3 School for Resource and Environmental Studies, Dalhousie University Halifax, Nova Scotia, Canada; University of Pennsylvania, United States of America

## Abstract

Global scale forecasts of range shifts in response to global warming have provided vital insight into predicted species redistribution. We build on that insight by examining whether local warming will affect habitat on spatiotemporal scales relevant to regional agencies. We used generalized additive models to quantify the realized habitat of 46 temperate/boreal marine species using 41+ years of survey data from 35°N–48°N in the Northwest Atlantic. We then estimated change in a “realized thermal habitat index” under short-term (2030) and long-term (2060) warming scenarios. Under the 2030 scenario, ∼10% of species will lose realized thermal habitat at the national scale (USA and Canada) but planktivores are expected to lose significantly in both countries which may result in indirect changes in their predators’ distribution. In contrast, by 2060 in Canada, the realized habitat of 76% of species will change (55% will lose, 21% will gain) while in the USA, the realized habitat of 85% of species will change (65% will lose, 20% will gain). If all else were held constant, the ecosystem is projected to change radically based on thermal habitat alone. The magnitude of the 2060 warming projection (∼1.5–3°C) was observed in 2012 affirming that research is needed on effects of extreme “weather” in addition to increasing mean temperature. Our approach can be used to aggregate at smaller spatial scales where temperate/boreal species are hypothesized to have a greater loss at ∼40°N. The uncertainty associated with climate change forecasts is large, yet resource management agencies still have to address climate change. How? Since many fishery agencies do not plan beyond 5 years, a logical way forward is to incorporate a “realized thermal habitat index” into the stock assessment process. Over time, decisions would be influenced by the amount of suitable thermal habitat, in concert with gradual or extreme warming.

## Introduction

Global scale meta-analyses of biological responses to climate change have provided vital insight into changes in distribution, phenology and species interactions over the last 2–3 decades [1]. Such information is necessary for policy makers working on general climate change adaptation strategies, but not sufficient for marine regulatory agencies where commercial species catches are highly regulated at smaller spatial and temporal scales. At regional and local scales (∼10–100 km^2^), the uncertainty in climate change projections increases [2] yet marine populations are highly responsive to regional temperature variation. It is indeed a challenge to provide regional scale information on how and when marine species will respond to climate change so that ocean managers can modify governance structures accordingly [3].

The biological response to climate variation can be complex but a population’s first acclimative response is typically a shift in spatial distribution in response to temperature change [1], [4]. In the ocean, global model projections predict that fish will gradually migrate poleward from 2005–2050 [5]. Recently, it has been shown that change is not necessarily poleward; there can be a variety of directional responses to local changes in temperature [6]. Range shifts on regional scales are already evident in the North Sea [7] and off the northeastern United States [8]. For a few commercial species on the US Northeastern seaboard, fisheries management strategies have lagged behind their northerly shifts [9]. In other areas the rate of temperature increase is slower [10] or well within the range of natural variability [11]. It has long been understood that temperature is a primary determinant of species distribution and we need to understand how species habitat availability will be affected by warming trends.

The Species Distribution Model (SDM) has been used in many studies to explore and forecast range shifts in response to warming. The SDM is based on the concept of an ecological “niche” where distribution is first and foremost determined by physiological constraints imposed by environmental variables (i.e. the “Fundamental” niche, reviewed in [12]). But an animal’s distribution is not just determined by environment; it is further constrained by species interactions such as competition and predation. This further constrained distribution has been referred to as the “Realized” niche. As such, one of the greatest weaknesses of most SDMs is that predictions do not account for species interactions, micro-evolutionary changes or dispersal abilities [13]. Most SDMs use a correlative approach whereas the much more difficult mechanistic approach can provide more insight and improve predictability [14], [15]. The uncertainty of correlative SDMs to predict species distribution is exacerbated by the uncertainty in climate change projections and the influence of other anthropogenic factors such as fishing and pollution [16]–[18]. Still, correlative SDMs can provide insight in appropriate situations and are constantly being modified to improve their application [18], [19].

**Figure 1:**
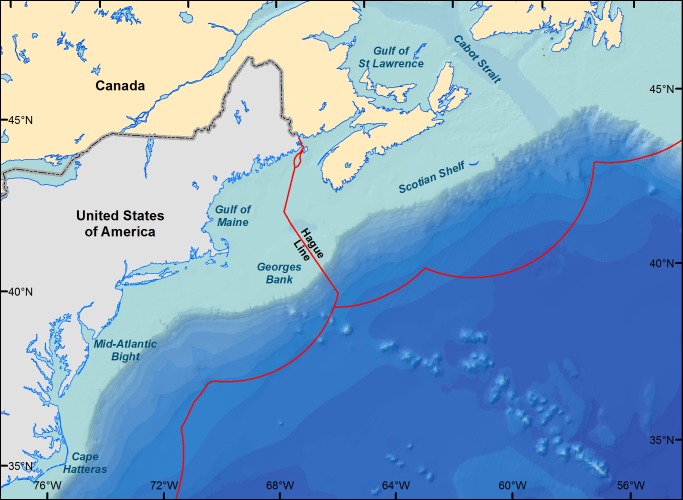
Northeast Continental Shelf (United States) and Scotian Shelf (Canada) in the Northwest Atlantic. The red line demarcates each country’s jurisdiction, separated by the Hague line. Most of the species studied are more common north of the Mid-Atlantic Bight.

In this study, we provide a broad regional-scale overview of changes in a realized thermal habitat index to projected short (2030) and long-term (2060) warming. We used a correlative SDM approach to quantify the realized habitat for each of 46 species using 41+ years of survey data from Cape Hatteras, North Carolina to Cape Breton Island, Nova Scotia, Canada (Figure 1). We estimated changes (and confidence limits) in the amount of each species’ realized thermal habitat index under short-term (2030) and long-term (2060) warming scenarios. Our goals are to provide an initial triage to gauge the severity of ecological/economic impacts and to flag vulnerable or expanding species in the USA and Canada. With such information, policy-makers and ocean managers could evaluate the risks of management action (or inaction) and use our approach to examine a subset of species at smaller spatial scales within the USA and Canada.

## Materials and Methods

### Data

Data used herein are publicly available from the Ocean Biogeographic Information System (OBIS) under the dataset names “DFO Maritimes Research Vessel Trawl Surveys Fish Observations” (OBIS-Canada) and the “Northeast Fisheries Science Center Bottom Trawl Survey Data” (OBIS-USA) [20], [21] (http://iobis.org/). Presence/absence data of 46 species (Table 1) from 35°N to 48°N from 1963–2012 were extracted (Figure 1). The species selected are common throughout the Gulf of Maine, Georges Bank and on the Scotian Shelf and form a temperate/boreal complex. We also obtained the environmental covariates of interest (date, depth, bottom temperature, latitude and longitude) from the OBIS datasets. Bottom temperature was recorded for each survey tow whether fish were present or not; these data are ideal for examining how local bottom temperature variation affects species distribution (Table S1).

**Table 1:** Generalized Additive Model (GAM) result summaries for each species using Full dataset and 10 subsamples.

Species	Scientific Name	AUC.Full	Deviance.Explained.Full	AUC.mean	AUC.sd	Deviance.Explained.mean	Deviance.Explained.sd	Num.SubSampleTrials
Barndoor_Skate	*Dipturus laevis*	0.95	0.95	0.0018	0.44	0.43	0.0063	10
Black Belly_Rosefish	*Helicolenus dactylopterus*	0.93	0.93	0.0025	0.41	0.41	0.008	10
Capelin	*Mallotus villosus*	0.98	0.98	0.0012	0.55	0.56	0.01	10
Cod	*Gadus morhua*	0.88	0.88	0.002	0.4	0.39	0.0047	10
Cunner	*Tautogolabrus adspersus*	0.89	0.88	0.0041	0.25	0.24	0.0078	10
Cusk	*Brosme brosme*	0.93	0.92	0.0032	0.38	0.37	0.0081	10
Dogfish	*Squalus acanthias*	0.8	0.8	0.0014	0.23	0.23	0.0016	10
Dory	*Zenopsis conchifera*	0.98	0.98	0.0068	0.43	0.45	0.041	10
Gulf Stream_flounder	*Citharichthys arctifrons*	0.9	0.9	0.0022	0.36	0.37	0.0063	10
Haddock	*Melanogrammus aeglefinus*	0.9	0.9	0.0028	0.41	0.41	0.0072	10
Hagfish	*Myxine glutinosa*	0.88	0.88	0.0042	0.26	0.26	0.0074	10
Halibut	*Hippoglossus hippoglossus*	0.89	0.89	0.0035	0.31	0.3	0.0077	10
Herring	*Clupea harengus*	0.84	0.84	0.0037	0.28	0.28	0.0066	10
Jonah Crab	*Cancer borealis*	0.83	0.83	0.0043	0.22	0.22	0.0059	10
Little_Skate	*Leucoraja erinacea*	0.91	0.91	0.0017	0.43	0.43	0.0054	10
Lobster	*Homarus americanus*	0.83	0.82	0.003	0.25	0.25	0.004	10
Longhorn_Sculpin	*Myoxocephalus octodecemspinosus*	0.88	0.88	0.0019	0.35	0.36	0.0043	10
Monkfish	*Lophius americanus*	0.76	0.76	0.0024	0.15	0.16	0.0026	10
Moustache_Sculpin	*Triglops murrayi*	0.89	0.88	0.0039	0.29	0.28	0.0082	10
Northern_Shrimp	*Pandalus borealis*	0.98	0.98	0.001	0.64	0.64	0.0075	10
Ocean_Pout	*Macrozoarces americanus*	0.83	0.82	0.0025	0.24	0.24	0.0041	10
Offshore_Hake	*Merluccius albidus*	0.95	0.95	0.0046	0.46	0.47	0.014	10
Plaice	*Hippoglossoides platessoides*	0.93	0.93	0.002	0.53	0.53	0.0067	10
Pollock	*Pollachius virens*	0.85	0.85	0.0022	0.28	0.28	0.004	10
Radiated Shanny	*Ulvaria subbifurcata*	0.86	0.87	0.012	0.16	0.18	0.013	10
Red_Crab	*Geryon quinquedens*	0.95	0.95	0.0044	0.4	0.4	0.021	10
Red_Hake	*Urophycis chuss*	0.81	0.81	0.002	0.23	0.23	0.003	10
Redfish	*Sebastes fasciatus*	0.94	0.94	0.0013	0.51	0.51	0.0059	10
Rock_Crab	*Cancer irroratus*	0.87	0.87	0.0029	0.32	0.31	0.0063	10
Sandlance	*Ammodytes dubius*	0.86	0.87	0.005	0.27	0.27	0.0094	10
Scallop	*Placopecten magellanicus*	0.86	0.86	0.0021	0.29	0.3	0.0048	10
Sea Raven	*Hemitripterus americanus*	0.83	0.83	0.0022	0.24	0.24	0.0039	10
Shortfin_Squid	*Illex illecebrosus*	0.82	0.83	0.0029	0.24	0.25	0.005	10
Silver_Hake	*Merluccius bilinearis*	0.82	0.83	0.0032	0.26	0.27	0.0049	10
Smooth_Skate	*Malacoraja senta*	0.87	0.87	0.0023	0.3	0.3	0.0051	10
Snow_Crab	*Chionoecetes opilio*	0.98	0.99	0.001	0.68	0.68	0.0082	10
Summer_Flounder	*Paralichthys dentatus*	0.92	0.92	0.0016	0.45	0.44	0.0046	10
Thorny_Skate	*Amblyraja radiata*	0.91	0.91	0.0019	0.44	0.43	0.0044	10
Turbot	*Reinhardtius hippoglossoides*	0.98	0.98	0.0027	0.6	0.61	0.02	10
White_Hake	*Urophycis tenuis*	0.91	0.91	0.0016	0.43	0.43	0.0048	10
Windowpane	*Scophthalmus aquosus*	0.91	0.91	0.0014	0.42	0.43	0.0036	10
Winter_Flounder	*Pseudopleuronectes americanus*	0.9	0.9	0.0023	0.41	0.4	0.0064	10
Winter_Skate	*Leucoraja ocellata*	0.86	0.86	0.0021	0.31	0.31	0.0042	10
Witch_Flounder	*Glyptocephalus cynoglossus*	0.87	0.86	0.003	0.32	0.32	0.0065	10
Wolffish	*Anarhichas lupus*	0.89	0.88	0.0036	0.31	0.3	0.0077	10
Yellowtail_Flounder	*Limanda ferruginea*	0.86	0.87	0.002	0.33	0.33	0.0041	10

AUC refers to Area under the Curve Statistic. The subscript “Full” refers to using all data whereas “mean” and “sd” refer to mean and standard deviation among subsamples.

### Model Approach

There are a variety of SDM approaches to explore possible shifts in species distribution and an associated branch of research that has evaluated the assumptions and methodologies among approaches [22]–[24]. We used these evaluations to select the most appropriate approach, given our data. For this broad exercise of examining multiple species, we used a correlative approach [14]. We opted to use a binomial Generalized Additive Model (GAM) using a logit link function [25] as the SDM. Our decision was based on reviews that showed GAMs were superior to other SDM techniques under circumstances similar to ours [24], [26]. For each species, presence/absence data were modeled as a function of the following covariates: location (latitude, longitude), year, bottom temperature, and depth. The fitted values were estimates of the probability of occurrence at location for each species and reflect “realized habitat”. In preliminary analyses of several common species, we fit models using only bottom temperature in efforts to estimate “Potential” thermal habitat, but concluded that the model over-predicted occurrences for the purpose of estimating change under warming scenarios. Therefore we conservatively defined model fit as a “Realized Thermal Habitat Index” to reflect the correlative nature of our SDM [14]. All analyses were conducted using the R statistical language [27] using various packages including the “mgcv” package [28]. Further details are provided in the Supporting Information.

### Model Evaluation

We evaluated each model’s accuracy using a method, similar in intent to the Wilcoxon Rank Sum test, called the “Area Under the Receiver Operating Characteristic Curve (AUC) available in R CRAN package “caTools”. This method evaluates how well the final model predicts a true positive and a true negative [29].

### Regional Warming Scenarios

Global climate models (GCM), developed from general circulation models, or earth system models often have limited application at smaller spatial scales, yet organisms respond to smaller-scale variation. Methods to down-scale from large-scale GCM coarse resolution (grid size∼100–300 km^2^) to regional/local scale resolution (grid size:∼5–100 km^2^) are still evolving rapidly [2] and are not yet fully available for the Canadian portion of our study domain. Accuracy of SDMs predictions are confounded by uncertainties in GCM climate forecasts and uncertain “down-scaling” [22]. As described earlier, the field of predicting species distribution from SDMs is evolving as well [30]. All SDM climate change analyses suffer from these compounding sources of uncertainty. We used the most recent information available [11], [31] to create 2 likely scenarios in which to evaluate projected change in realized habitat indices.

The two scenarios are derived from regional syntheses of sea surface temperature (SST) projections [11] and trends [31] for the Scotian Shelf and Gulf of Maine. These trends and projections are for SST. Reliable bottom temperature projections are not yet available but we recognize that deeper waters warm more slowly than surface water. Therefore, the first scenario is derived from a long-term (50–60years) mid-range projection (3°C) from an ensemble of AR5 ESM August SST projections for the region [11]. Using each species dataset, we added 3°C to all bottom temperature data collected at depths equal to or less than 100 m, and 1.5°C to depths more than 100 m. The second scenario is based on SST empirical decadal trends in the region since 1985 [31] adjusted for 20 years (0.7°C added to bottom temperature at depths equal to or less than 100 m, and 0.35°C added to depths more than 100 m). We refer to Scenario 1 as Y2060 and Scenario 2 as Y2030.

### Estimating Gain/Loss of Realized Thermal Habitat Index

We estimated area of habitat where probability of occurrence from the original model output was more than 0.54. Note that we could have selected any habitat >0.5 as our goal was to be conservative and estimate the change in the most *likely* realized thermal habitat index. We did the same for each of the projected scenarios’ model output (e.g. Y2060 and Y2030 realized thermal habitat index). Net change (gain/loss) was the percentage of current realized thermal habitat index less the future, divided by the current realized thermal habitat index.

The common method of sample-splitting data (using one set to calibrate a model and the other to test) does not address whether a model is suitable for extrapolation [24]. Further, Araújo et al. [32] argued that splitting the data into 2 sets does not provide an independent test as both sets are derived from the same source. However, we did wish to gauge the sample error in the gain/loss estimate. The range of estimates for each species is an index of uncertainty, which in turn discerns whether our method is appropriate for a given species. A large range would indicate that this method was not appropriate for the species, and other research avenues would have to be pursued. Therefore, for each species, we fit GAMs to 10 random sub-samples (0.6 of full dataset). This provided a range of all model output but more importantly a range of the estimated gain/loss as calculated based on probability estimates.

## Results

### Model Evaluation

**Figure 2:**
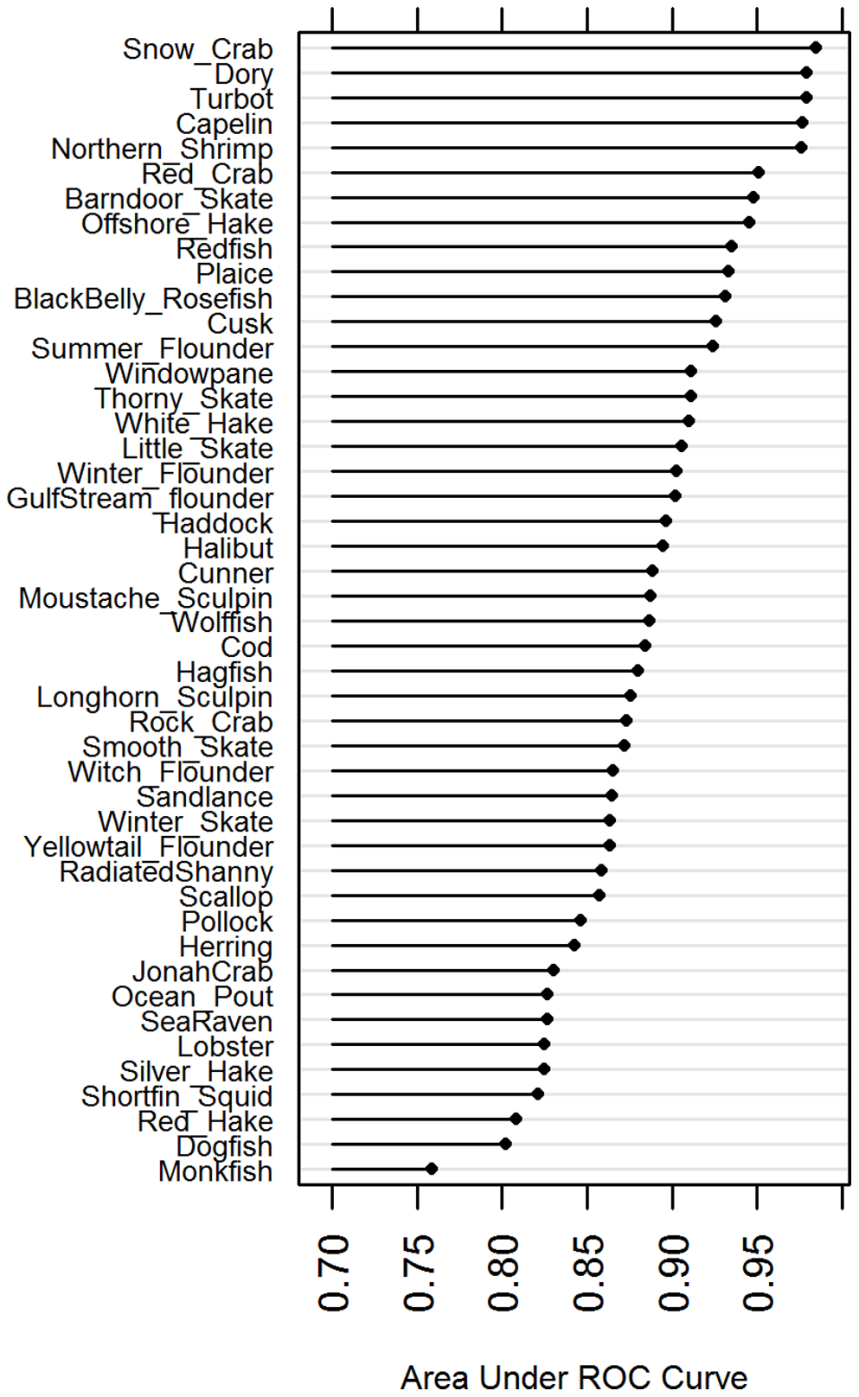
Area Under Receiving Operating Curve (AUC). Expected Accuracy (of classification) of GAM model fit for each species, compared to the observations. An AUC value of 1 would indicate that predicted values resulted in a completely accurate classification of observations. For all but one species, the model was able to accurately classify >80% probability of occurrence.

Accuracy of predictions among models for 46 species, ranged from 0.77–0.99 (Median = 0.89) indicating strong accuracy for all species (Figure 2, Table 1). The power is derived from 41+ years of observations over a large geographic scale (∼35°N–48°N) and spanning a wide temperature range (∼–1.5°C to 25°C). While the models accurately reproduce the probability of occurrence for all species, it is evident that they are more accurate, and a higher amount of deviance is explained, for those species that are restricted by warmer temperatures throughout the study domain (Table 1). Snow crab, capelin, turbot, and northern shrimp would all be considered to be at the southern limit of their distribution within this study domain(∼41°N), indicating that the many zero values in the southern part of the domain (<41°N) contributed to increased accuracy.

### Between Countries and Scenarios

**Figure 3:**
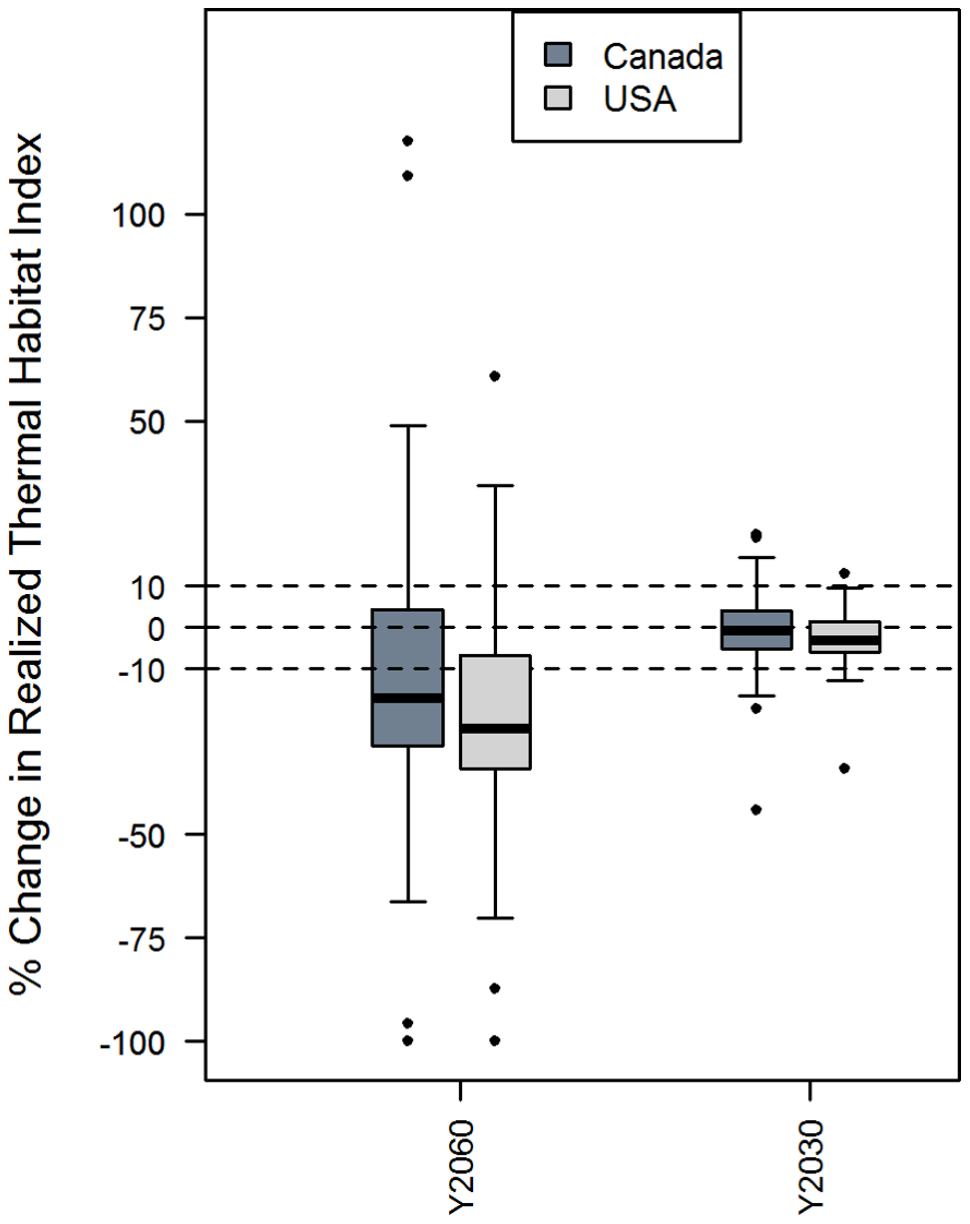
Change in Realized Thermal Habitat Index among species within Canada and USA. Boxplots represent distribution of net change among species within nation under the long-term (Y2060) and short-term (2030) scenarios.

The median net change in the realized thermal habitat index is similar between countries and scenarios. Under the long-term (2060) warming scenario (Scenario 1), the majority of species in Canada and in the USA will lose realized thermal habitat (Figure 3). Under the short term (2030), Scenario 2, the median loss of both nations is neutral (within +/–10%). There are a few outliers indicating that there are extreme winners and losers, regardless of whether the majority stay relatively neutral (Figure 3). The majority of species are neutral as a result of the northern temperate/boreal nature of the species examined, and the scale of aggregation at the national level. It is important to note that there will be a more pronounced response for some species at smaller spatial scales. When the domain is divided into sub-regions, there can be a stronger negative response in the USA and on south-west Scotian shelf for species that are at the southern limit of their range (See Figure S1 for an example of cod’s response at sub-regional scales).

### Ecological perspective

Trophic balance among functional groups contributes to ecosystem stability. Overfishing of one functional group can result in an explosion of their prey and competitors and lead to trophic imbalance [33]. Colder less diverse systems succumb more quickly to overfishing, and take longer to recover due to trophic imbalance than warmer, species rich regions [34]. From an ecosystem perspective, we examined whether one functional group is forecast to lose more realized thermal habitat than another, which would exacerbate any imbalance. Functional group membership for each species is listed in Table S2 and are derived from Shackell et al [35].

**Figure 4:**
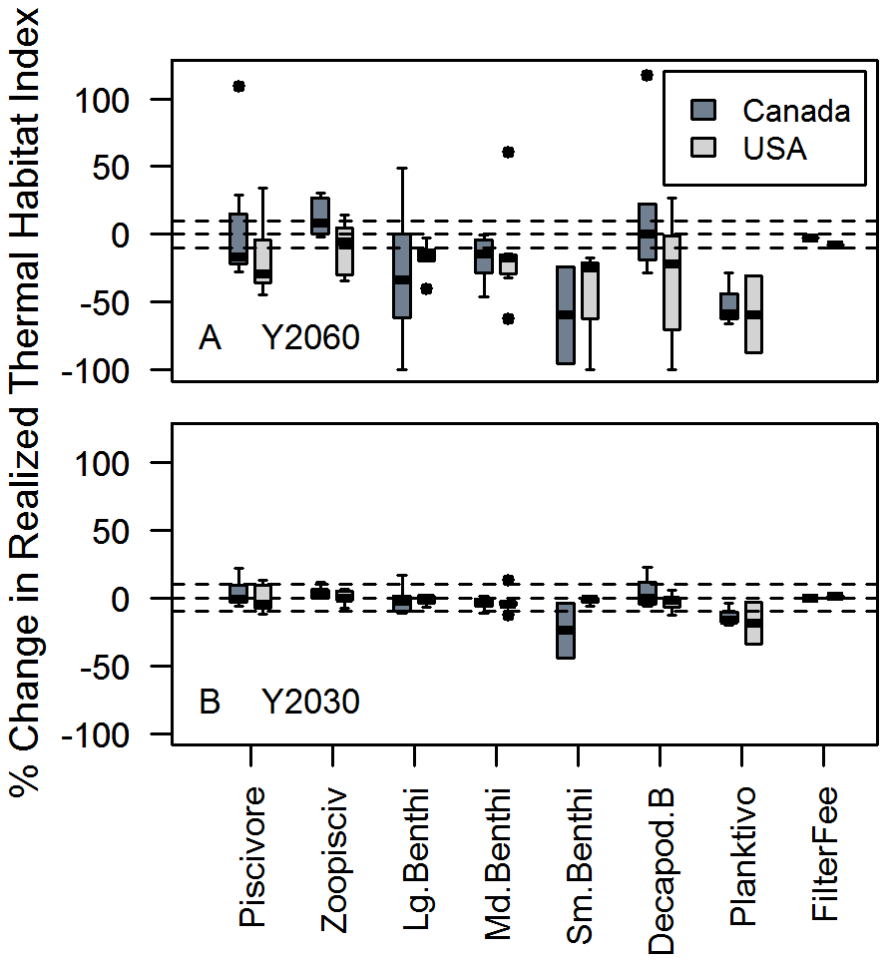
Change in Realized Thermal Habitat Index by functional group in Canada and USA. Panels depict results under the long-term (Y2060) (A) and short-term (Y2030) (B) scenarios. Functional group member species are listed in Table S1. Piscivore refers to piscivore. Zoopisciv refers to zoopiscivore, Lg.Benthi refers to large benthivores, Md.Benthi refers to Medium-sized benthivores, Sm.Benthi refers to small-sized benthivores. Decapod B refers to decapod benthivores, Planktivo refers to planktivores, FilterFee refers to filter feeders (scallop).

From an ecological perspective, in 2060 (Scenario 1) in Canada, functional groups differed significantly, according to a rank-sum test (Kruskal-Wallis chi-squared  =  15.3, df  =  6, p =  0.02) but not in the USA (Kruskal-Wallis chi-squared  =  4, df  =  6,p  =  0.7) (Figure 4A). Both nations follow a similar pattern, yet the difference is that small benthivores are expected to lose more realized thermal habitat in Canada than in the USA. The net change in planktivores (sandlance, herring, capelin) is more negative in both nations than piscivores; significantly so in Canada (Kruskal-Wallis chi-squared  =  6.8, df  = 1, p<0.01) but not in the USA (Kruskal-Wallis chi-squared  =  1.2, df  =  1, p  =  0.3).

Differences among functional groups are not significant in Scenario 2 in Canada (Figure 4B) (Kruskal-Wallis chi-squared  =  12.12, df  =  6, p  =  0.06), nor in the USA: (Kruskal-Wallis chi-squared  =  3.1, df  =  6, p-value  =  0.8) (Figure 4B). Under a weak warming scenario, planktivores uniformly lose more realized thermal habitat than other functional groups, but this does not amount to a statistically significant difference.

### Economic perspective

**Figure 5:**
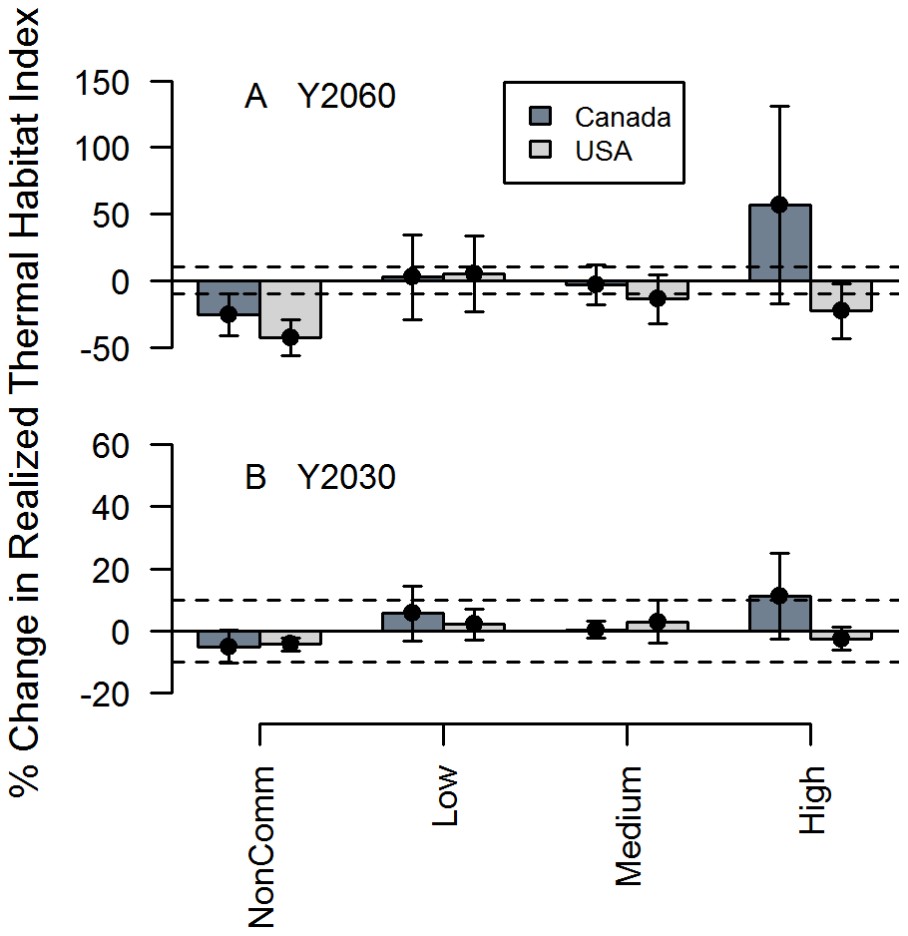
Change in Realized Thermal Habitat Index by commercial category in Canada and USA. Panels depict results under the long-term (Y2060) (A) and short-term (Y2030) (B) scenarios. Commercial categories were defined by quantiles of 2011commercial value estimated separately for Canada and USA. The y-axis represents the weighted mean where the “Change in realized thermal habitat” index was weighted by species commercial value within categories. Values for each country were derived separately from 2011 commercial landing and value statistics for USA( http://www.st.nmfs.noaa.gov/commercial-fisheries/commercial-landings/monthly-landings/index) and Canada (http://www.dfo-mpo.gc.ca/stats/commercial/land-debarq/sea-maritimes/s2011av-eng.htm) and used to calculate weighted mean and confidence intervals.

How will the change in the realized thermal habitat index affect non-commercial and commercial species? As an exercise, we weighted net change of the realized thermal habitat index by the 2011 value of landings in categories of non-commercial, low, medium and highly commercial species separately for Canada and the USA (Table S1). By 2060, currently highly commercial species in Canada would gain realized thermal habitat, while USA species of the same category would lose (Figure 5). In 2030, results are similar but dampened. These patterns are driven by lobster which was an order of magnitude of greater value than other highly valued species in Canada (scallop, snow crab) in 2011 (Table S1). Non-commercial species will lose realized thermal habitat while species of low commercial value will gain in the short and long-term scenarios in both countries. There is very little change in the medium commercial category in the long term, while in the short term, these species will gain realized thermal habitat in the USA.

### Species Perspective

Which species will gain or lose significant realized thermal habitat? The national, economic and ecological perspectives above provide overviews of the system whereas species-level information might arguably be used most by managers to set priorities. To lessen uncertainty in the forecast, we estimated net change in the realized thermal habitat index based solely on the most likely habitat, as defined by the probability estimate from the original model of >0.54 likelihood. In addition, we ran the model 10 times on subsamples (60%) to gauge the error surrounding net change in the realized thermal habitat index. In this manner, decisions can be based on level of certainty of results. By our definition, the original full model did not estimate highly probable habitat for the following species in the following regions: radiated shanny, cunner, and dory in Canada and USA, summer flounder in Canada, capelin, halibut, moustache sculpin and wolfish in the USA. Consequently, these species were not included in habitat gain/loss predictions for the relevant regions.

**Figure 6:**
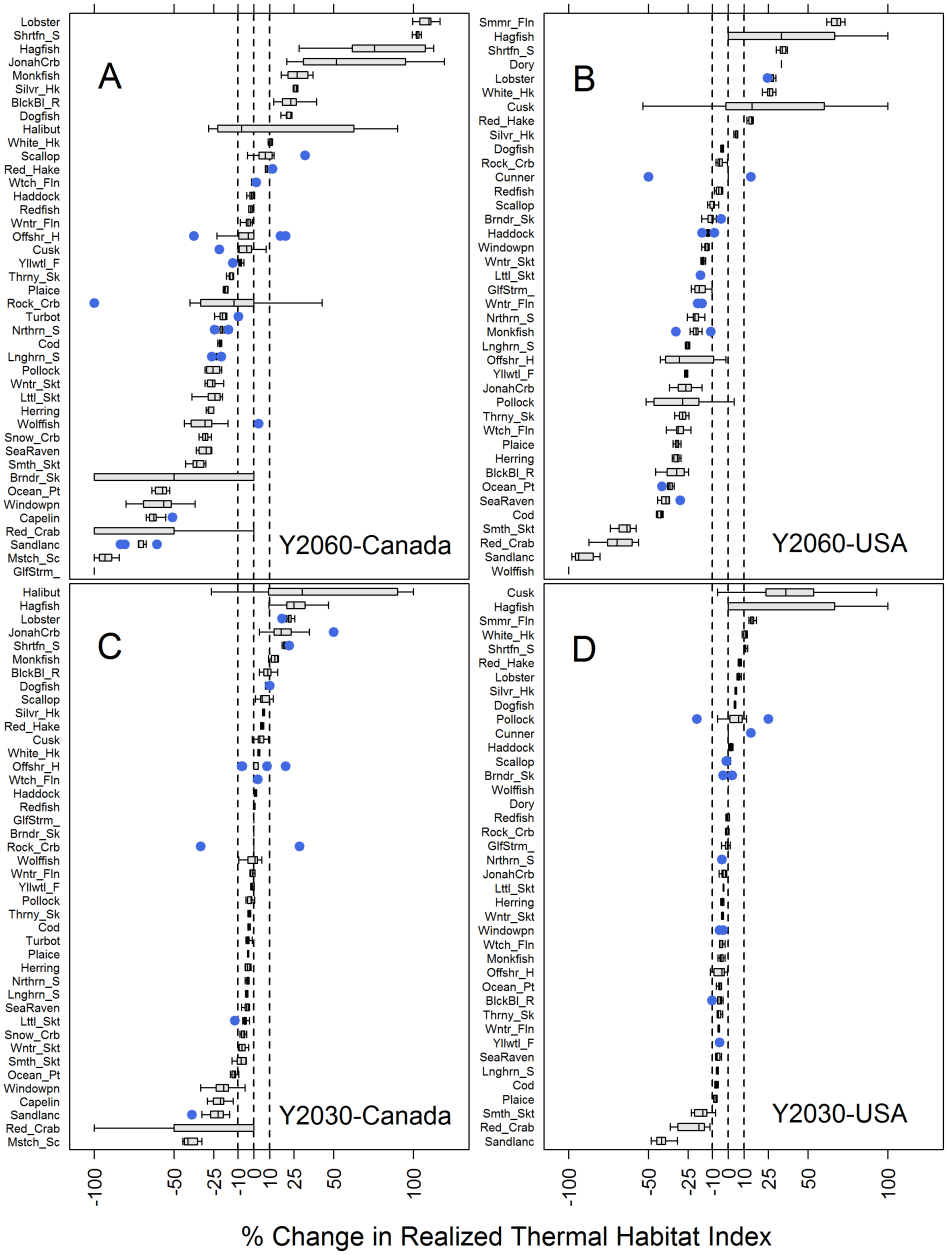
Change in Realized Thermal Habitat Index of individual species for long-term (2060) and short-term (2030) scenarios . Each boxplot represents the distribution of net change calculated from 10 models fit to subsamples of each species. Dashed lines are at 10%. Net change is considered neutral if median net change among 10 subsamples is between dashed lines. Positive outliers >100 are not shown. Change in habitat under scenario 1 (Y2060) in Canada (A) and the USA (B) and under scenario 2 (Y2030) in Canada (C) and USA (D). Abbreviated species names are listed beside full common names in Table S2.

By 2060, 55% of the species examined will lose realized thermal habitat (<– 10%), 21% will gain (>10%), while 24% will stay neutral in Canada (Figure 6A). In the USA, 65% of the species will lose realized thermal habitat (<–10%), 20% will gain (>10%), while 15% will stay neutral (Figure 6B). By 2030, 12% of the species examined will lose realized thermal habitat (<– 10%), 14% will gain (>10%), while 74% will stay neutral in Canada (Figure 6C). By 2030, in Canada, losers with reasonable error bars include: moustache sculpin, sandlance, capelin, windowpane, ocean pout, while winners include: hagfish, lobster, jonah crab, shortfin squid, and monkfish. By 2030, in the USA, 8% of the species will lose realized thermal habitat (<– 10%), 10% will gain (>10%) while 83% will stay neutral (Figure 6D). By 2030, in the USA, losers include: sandlance, red crab and smooth skate while winners include: summer flounder, white hake and shortfin squid.

## Discussion

We have provided a broad overview of how projected warming may affect species realized thermal habitat indices on the US Northeast Continental and Scotian Shelves. We narrowed down the most likely scenarios on a regional-scale and then provided an overview of the expected response to warming from national, ecological, economic and species perspectives. We have built on previous global-scale studies by taking the necessary step of analyzing finer-scale biological and oceanographic data [6]. On a regional scale (USA and Canada), most species realized thermal habitat will withstand warming projected for 2030, but not for 2060. Importantly, the magnitude of the 2060 warming projection (∼1.5–3C) was observed in 2012 across the northern part of the study domain [36]. We did not focus on the effect of current extremely anomalous years but it would be useful to pursue how species respond to extreme events in that it may affect distribution and productivity [37], [38] and ultimately local economies.

Populations may respond initially through a change in distribution, but over the long term, changes in productivity, predation, competition, species adaptations and fishing pressure will undoubtedly play much larger roles than warming [5], [16], [39], [40]. The long-term Scenario 1 (Y2060) results enabled us to gauge model performance, the extent that the community will change over time, and have raised the question of how current extreme events will affect productivity, and whether we should expect more anomalously warm years under climate change. Our broad overview on a regional scale showed that, under the more realistic short-term (Scenario 2: Y2030), only a few species are expected to lose realized habitat in both Canada and the USA, but this should be considered a starting point. As northern temperate/boreal species lose habitat, other species not considered in this analysis, including currently more southerly distributed species, will fill empty niches. This effect will be most pronounced in the Gulf of Maine/Georges Bank area where many temperate/boreal species are at the southern limit of their range. Initial analyses show that on smaller sub-regional scales, there is considerably more spatial variation in response where southerly distributed cod populations lose more realized thermal habitat than in the northeasterly subregions (Figure S1). Our approach can easily be used to aggregate at smaller spatial scales and this is an area of future research.

Planktivores, a key part of this region’s food chain, are expected to lose significant realized thermal habitat in both countries; this has implications for all species that eat planktivores. That is, changes in distribution and productivity of prey could result in indirect changes in predator distribution and productivity. However, planktivores, such as herring, spend only part of their time near the bottom and they are not caught as easily as groundfish by the surveys used in this analysis. To assess the potential direct and indirect impacts of warming on distribution and trophic balance, a next step, which is beyond the scope of this study, could be to investigate planktivore catchability. This would, of course, require accurate catchability indices for all species for each survey.

### SDM as a Decision-making Tool: Caveats

Regarding those species whose expected net change is not neutral (more or less than 10%), it is important that any interpretation accounts for two issues, 1) the confidence of the net change estimate, and 2) the current extent of the species distribution. First, the range of net change among up to 10 models per species is an indication of response prediction uncertainty. When the range of values is large, definitive statements cannot be made and managers would have to seek further corollary information on the species of interest. In this analysis, those species would include: hagfish, jonah crab, halibut, barndoor skate, red crab and cusk in either USA or Canada. The first step towards improving these species estimates would be to aggregate results for each species on smaller spatial scales, and to seek and examine other environmental covariates that may be important to the species. Cusk, for example, are associated with terrain complexity and may not even be catchable on highly complex untrawlable bottom [41]. Hare et al. [41] modelled cusk habitat and included a covariate important to cusk: terrain ruggedness. While their model is not directly comparable to ours because they defined a broader window of “potential” habitat, their study illustrates the benefits of focussing on a single species.

Even when the sample error suggests confidence in our forecast, corollary information on the species of interest should be pursued. Sandlance is an expectant loser common across both scenarios and in both countries (Figure 6). Even though confidence is high, further research should be pursued to corroborate or qualify results as sandlance is an important forage fish.

**Figure 7:**
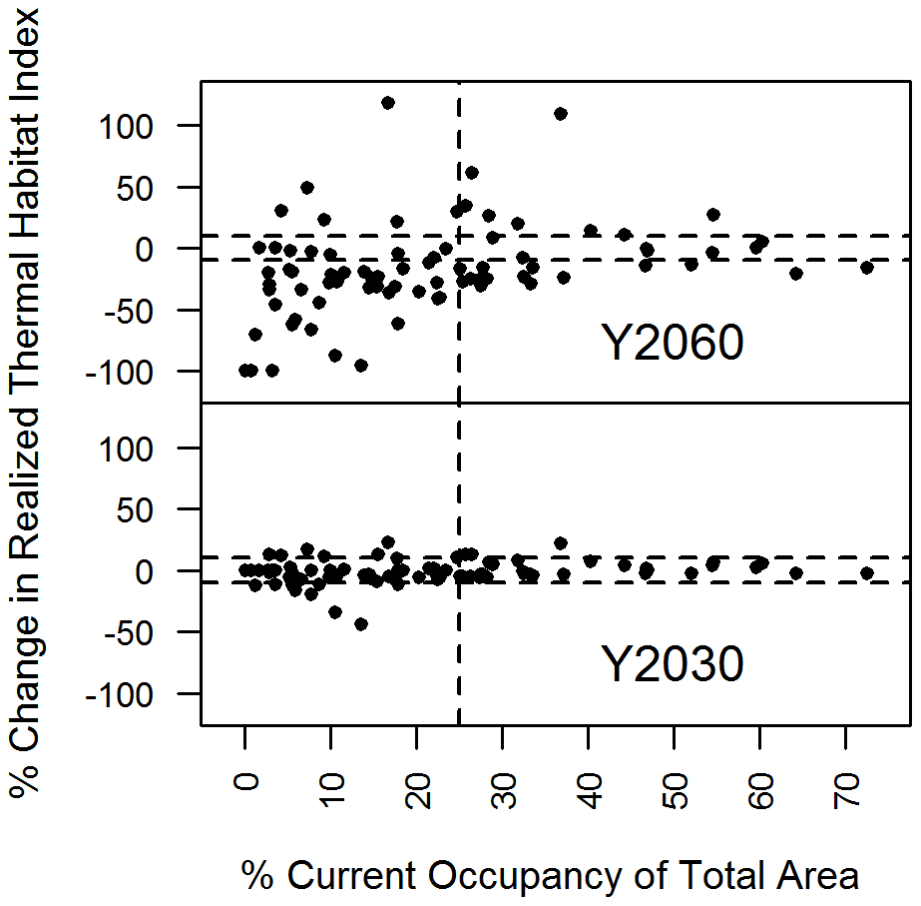
Change in Realized Thermal Habitat Index habitat and current occupancy. Lower rates of change are expected for widespread animals but the effect is only noticeable under Y2060 higher average temperatures or extreme events, as occurred in 2012.

Second, there is a positive relationship between abundance and distribution [42]. Widespread animals are generally more adaptable and can withstand a greater range of environmental conditions. There was no relationship between change in realized thermal habitat and current extent of distribution on the short-term, but when the thermal envelope is shifted enough, as in the long-term scenario, the change was only neutral for widespread species, whose extent of distribution is more than ∼22% of the total area (Figure 7). In effect, widespread species have a greater thermal window in the study domain. We might also expect that less widespread species will have a greater negative response in anomalously warm years. The relationship between net change and distribution (Figure 7) also provides context to the impact of a large net change; the realized thermal habitat index for a species may be projected to decrease by 75%, but the current total area can be quite small.

Our approach is flexible for managers. We arbitrarily designated +/–10% net change in the realized thermal habitat index as a window of neutrality. A fishery manager focused on depleted, threatened or endangered species may consider that window too narrow whereas those focussed on an increasingly abundant species would consider the window too broad. In either case, auxiliary information should always be used to provide context. Several species considered herein are currently depleted but were historically important ecological and commercial species. Some of these species have not fully recovered their size structure or former biomasses [43], [44] which means they are even more susceptible to climate variability [45]. It is probable that in 20 years, depleted species on the southern limit of their range will be extirpated from the USA as has been observed for terrestrial species [46]. In other words, the forecast of net change must be interpreted in the context of the species; extirpation will likely occur earlier for unhealthy populations.

### SDM as a Decision-making Tool: Direction

In our view, forecast uncertainty is not unique to SDMs in climate change research and that uncertainty will not diminish fast enough for practical application. Natural resource management agencies still have to tackle how climate change will affect their ability to safeguard resources. Twenty and 50 year forecasts derived from SDMs are insightful, but are they useful to fishery agencies who plan on much shorter time scales? We echo the sentiment of others [47] by suggesting that an imminent and promising use of SDMs is to incorporate a thermal habitat indicator into current stock assessments. We also suggest this would be relatively easy to implement. There has been considerable discussion on, and progress towards, “Ecosystem-based Management” over the last 25 years (reviewed in [48]). Many of the “Ecosystem Indicators” are climate–related, such as bottom temperature and sea surface temperature, and are routinely monitored by many nations. It follows that oceanographic information gathered for ecosystem-based management can be used to incorporate climate change directly into stock assessments by, for example, using the realized thermal habitat index. Climate projections are uncertain partly because natural variability often swamps the signal of gradual change. A practical approach would be to adjust quotas based on amount of thermal habitat either solely or in a production model [49]. Over time, quotas would be adjusted up or down, in concert with gradual climate change. In fact, a “realized thermal habitat index” should be incorporated into stock assessments if only to monitor the effect of extreme events on subsequent productivity.

It is common to use a risk management approach when dealing with climate adaptation issues; priority issues are identified and auxiliary information is sought when uncertainty is high. SDMs from ecological, economic and species level perspectives as presented here can be used as a starting point to develop fuller assessments and adaptation strategies to counteract climate change impacts [50]. Habitat availability may not be the climate change “bottleneck” for common marine benthic species in the Northwest Atlantic in the next 20 years but the effect of current extremely warm years is unknown. To that end, we can now ask harder questions: will extreme warming events become more frequent? How will change in the base of the food chain affect overfished populations? Forecasts derived from SDMs may be only a starting point, but are necessary and practical as has been demonstrated for other marine species [51]. SDMs can provide context for a larger vulnerability assessment that would include some estimate of productivity response, diet availability, other important climate drivers such as dissolved oxygen and pH [52] and/or the inclusion of current stressors that would affect or weaken the ecosystem’s ability to respond [53].

## Supporting Information

Figure S1 **Percent change in realized habitat index for cod (** ***Gadus*** **morhua) in 6 subregions.** Subregions are defined in Shackell et al 2012 [1] and describe ecoregions from Cape Breton Canada to Cape Hatteras, USA. MAB = Mid-Atlantic Bight, SNE = Southern New England, GB = Georges Bank, BoF = Bay of Fundy, WSS  = Western Scotian shelf, ESS = eastern Scotian Shelf (see Figure 1 in[1]). No estimates were made for MAB where amount of cod most likely habitat was too low.
(DOCX)

Table S1 **Summary of bottom temperature records**. Mean, standard deviation (SD), minimum (min) and maximum (max) bottom temperature and number of sets within month, year and region using cod (*Gadus morhua)* sub-file as an example.
(DOCX)

Table S2 **Summary of Species, Functional Groups, Commercial Categories, Name abbreviations**. Net change in Realized Thermal Habitat Index in Scenario 2 is also shown. NA refers to not enough probable habitat (insufficient probability estimates >0.5).
(DOCX)

File S1 **Supplementary Information on Surveys and Analyses.**
(DOCX)
