# Supplementary material for: Thermal Habitat Index of Many Northwest Atlantic Temperate Species Stays Neutral under Warming Projected for 2030 but Changes Radically by 2060
Source: PLoS One. 2014 Mar 5;9(3):e90662. doi: 10.1371/journal.pone.0090662 (PMC3944076; doi:10.1371/journal.pone.0090662)
Supplement: Figure S1 — Percent change in realized habitat index for cod ( Gadus morhua) in 6 subregions. Subregions are defined in Shackell et al 2012 [1] and describe ecoregions from Cape Breton Canada to Cape Hatteras, USA. MAB = Mid-Atlantic Bight, SNE = Southern New England, GB = Georges Bank, BoF = Bay of Fundy, WSS = Western Scotian shelf, ESS = eastern Scotian Shelf (see Figure 1 in[1]). No estimates were made for MAB where amount of cod most likely habitat was too low. (DOCX) [file pone.0090662.s001.docx]

**Figure S1. Percent change in realized habitat index for cod (*Gadus* morhua) in 6 subregions.**

Subregions are defined in Shackell et al 2012 [1] and describe ecoregions from Cape Breton Canada to Cape Hatteras, USA. MAB=Mid-Atlantic Bight, SNE=Southern New England, GB=Georges Bank, BoF=Bay of Fundy, WSS =Western Scotian shelf, ESS=eastern Scotian Shelf (see Figure 1 in[1]). No estimates were made for MAB where amount of cod most likely habitat was too low.

1. Shackell NL, Bundy A, Nye JA, Link JS (2012) Common large-scale responses to climate and fishing across Northwest Atlantic ecosystems. ICES J Mar Sci 69: 151–162. doi:10.1093/icesjms/fsr195.
