# Supplementary material for: Thermal Habitat Index of Many Northwest Atlantic Temperate Species Stays Neutral under Warming Projected for 2030 but Changes Radically by 2060
Source: PLoS One. 2014 Mar 5;9(3):e90662. doi: 10.1371/journal.pone.0090662 (PMC3944076; doi:10.1371/journal.pone.0090662)
Supplement: Table S1 — Summary of bottom temperature records. Mean, standard deviation (SD), minimum (min) and maximum (max) bottom temperature and number of sets within month, year and region using cod (Gadus morhua) sub-file as an example. (DOCX) [file pone.0090662.s002.docx]

**Table S1. Summary of bottom temperature records**. Mean,standard deviation (SD), minimum (min) and maximum (max) bottom temperature and number of sets within month, year and region using cod (*Gadus morhua)* subfile as an example.

| Region | YEAR | MONTH | Bottom Temperature-Mean | SD | Min | Max | Number of survey sets |
| --- | --- | --- | --- | --- | --- | --- | --- |
| CAN | 1963 | 7 | 6.74 | 1.22 | 4.2 | 8.9 | 52 |
| CAN | 1963 | 11 | 6.48 | 1.10 | 4.7 | 9.9 | 37 |
| CAN | 1964 | 1 | 5.24 | 1.33 | 2.2 | 6.9 | 55 |
| CAN | 1964 | 8 | 5.62 | 1.47 | 2 | 8.4 | 43 |
| CAN | 1964 | 11 | 5.71 | 1.13 | 2.6 | 7.7 | 38 |
| CAN | 1965 | 2 | 3.91 | 1.16 | 1.7 | 5.7 | 46 |
| CAN | 1965 | 7 | 5.00 | 1.22 | 1.5 | 7.8 | 53 |
| CAN | 1965 | 10 | 5.88 | 1.15 | 3.8 | 8 | 42 |
| CAN | 1966 | 1 | 4.77 | 1.20 | 3 | 6.2 | 33 |
| CAN | 1966 | 2 | 3.59 | 1.67 | 1.8 | 7.1 | 17 |
| CAN | 1966 | 10 | 5.75 | 1.63 | 2.2 | 10.2 | 35 |
| CAN | 1967 | 11 | 5.82 | 1.20 | 3.6 | 7.9 | 29 |
| CAN | 1967 | 12 | 5.75 | 0.35 | 5.5 | 6 | 2 |
| CAN | 1968 | 3 | 4.40 | 1.84 | 2.1 | 8 | 24 |
| CAN | 1968 | 4 | 5.76 | 2.53 | 1.8 | 9.6 | 15 |
| CAN | 1968 | 5 | 6.32 | 0.58 | 5.3 | 6.8 | 6 |
| CAN | 1968 | 11 | 7.77 | 1.68 | 4.9 | 10.9 | 44 |
| CAN | 1969 | 3 | 5.33 | 1.61 | 4.1 | 8.3 | 7 |
| CAN | 1969 | 4 | 5.44 | 1.50 | 2.1 | 8 | 29 |
| CAN | 1969 | 7 | 5.48 | 1.59 | 3.1 | 7.8 | 18 |
| CAN | 1969 | 8 | 7.28 | 0.88 | 5.5 | 10.1 | 38 |
| CAN | 1969 | 11 | 7.49 | 1.53 | 3.6 | 9.9 | 50 |
| CAN | 1970 | 3 | 6.06 | 1.71 | 2.3 | 8.7 | 19 |
| CAN | 1970 | 4 | 5.53 | 1.88 | 1.9 | 9.9 | 38 |
| CAN | 1970 | 7 | 5.31 | 1.99 | 1.18 | 11.79 | 134 |
| CAN | 1970 | 11 | 6.92 | 1.74 | 2.3 | 9.8 | 51 |
| CAN | 1971 | 4 | 5.22 | 1.78 | 1.4 | 8.5 | 57 |
| CAN | 1971 | 6 | 7.71 | 1.15 | 6.45 | 9.42 | 9 |
| CAN | 1971 | 7 | 5.70 | 2.26 | 1.5 | 10.51 | 101 |
| CAN | 1971 | 11 | 8.11 | 1.67 | 4.7 | 10.9 | 51 |
| CAN | 1972 | 4 | 6.33 | 2.08 | 2.4 | 10 | 56 |
| CAN | 1972 | 6 | 7.17 | 1.92 | 3.58 | 11.34 | 56 |
| CAN | 1972 | 7 | 4.74 | 2.66 | -0.34 | 10.93 | 88 |
| CAN | 1972 | 11 | 8.09 | 1.27 | 4.8 | 10 | 79 |
| CAN | 1973 | 5 | 6.81 | 2.37 | 1.9 | 13.2 | 70 |
| CAN | 1973 | 7 | 5.98 | 2.83 | 0.48 | 11.8 | 117 |
| CAN | 1973 | 8 | 3.13 | 1.67 | 0.89 | 5.2 | 14 |
| CAN | 1973 | 11 | 8.76 | 1.95 | 5.4 | 13.7 | 70 |
| CAN | 1974 | 4 | 7.14 | 2.18 | 4 | 11.8 | 22 |
| CAN | 1974 | 5 | 5.42 | 0.99 | 4.4 | 7.4 | 9 |
| CAN | 1974 | 7 | 6.10 | 2.99 | 0.57 | 12.11 | 137 |
| CAN | 1974 | 8 | 3.06 | 1.67 | 0.6 | 5.31 | 15 |
| CAN | 1974 | 10 | 8.69 | 1.81 | 7.2 | 12.5 | 10 |
| CAN | 1974 | 11 | 9.90 | 1.97 | 5.5 | 14.2 | 67 |
| CAN | 1975 | 4 | 4.73 | 2.34 | 2.3 | 8.6 | 14 |
| CAN | 1975 | 5 | 6.75 | 1.33 | 4.3 | 8.5 | 19 |
| CAN | 1975 | 7 | 6.66 | 2.47 | 1.81 | 12.6 | 94 |
| CAN | 1975 | 8 | 2.83 | 2.05 | 0.31 | 7.24 | 48 |
| CAN | 1975 | 10 | 7.07 | 1.39 | 5.4 | 9.2 | 6 |
| CAN | 1975 | 11 | 8.17 | 1.74 | 3.8 | 11.5 | 72 |
| CAN | 1976 | 4 | 6.90 | 1.44 | 4.5 | 10.2 | 62 |
| CAN | 1976 | 5 | 6.57 | 1.01 | 4.7 | 8.2 | 11 |
| CAN | 1976 | 7 | 7.54 | 2.45 | 1.35 | 11.02 | 98 |
| CAN | 1976 | 8 | 4.16 | 2.64 | 1 | 9.78 | 37 |
| CAN | 1976 | 11 | 9.74 | 1.49 | 7.5 | 13.3 | 47 |
| CAN | 1977 | 5 | 5.43 | 1.23 | 3.6 | 8.5 | 55 |
| CAN | 1977 | 7 | 6.46 | 2.40 | 1.05 | 10.94 | 143 |
| CAN | 1977 | 8 | 6.39 | 1.14 | 4.8 | 8.6 | 27 |
| CAN | 1977 | 11 | 7.21 | 2.12 | 2.8 | 10.1 | 51 |
| CAN | 1977 | 12 | 8.60 | 1.25 | 6.1 | 10.1 | 25 |
| CAN | 1978 | 4 | 3.73 | 0.30 | 3.4 | 4.1 | 4 |
| CAN | 1978 | 5 | 5.33 | 1.66 | 1.5 | 10 | 89 |
| CAN | 1978 | 7 | 5.70 | 2.25 | 1.27 | 10.25 | 141 |
| CAN | 1978 | 8 | 6.41 | 1.44 | 4.4 | 9.1 | 18 |
| CAN | 1978 | 10 | 8.10 | 1.41 | 5.7 | 10.6 | 43 |
| CAN | 1978 | 11 | 6.80 | 2.00 | 2 | 9.9 | 91 |
| CAN | 1978 | 12 | 5.63 | 2.13 | 1.6 | 9.3 | 29 |
| CAN | 1979 | 3 | 4.40 | 3.18 | -0.91 | 9.99 | 115 |
| CAN | 1979 | 4 | 4.95 | 1.44 | 3.2 | 7.8 | 35 |
| CAN | 1979 | 5 | 5.23 | 1.23 | 3.2 | 7.6 | 26 |
| CAN | 1979 | 6 | 6.50 | NA | 6.5 | 6.5 | 1 |
| CAN | 1979 | 7 | 6.22 | 2.67 | 0.57 | 13.63 | 145 |
| CAN | 1979 | 8 | 6.98 | 1.13 | 5.8 | 8.6 | 6 |
| CAN | 1979 | 10 | 6.44 | 3.17 | 1.57 | 13.64 | 76 |
| CAN | 1979 | 11 | 8.39 | 1.93 | 4.28 | 13.13 | 130 |
| CAN | 1980 | 3 | 4.98 | 3.20 | 0.11 | 10.75 | 104 |
| CAN | 1980 | 4 | 4.53 | 0.77 | 3.1 | 5.4 | 9 |
| CAN | 1980 | 5 | 5.58 | 1.13 | 3.9 | 8.9 | 37 |
| CAN | 1980 | 7 | 6.17 | 2.40 | 1.27 | 10.31 | 145 |
| CAN | 1980 | 8 | 7.12 | 1.38 | 5.3 | 9.1 | 14 |
| CAN | 1980 | 9 | 9.70 | NA | 9.7 | 9.7 | 1 |
| CAN | 1980 | 10 | 6.42 | 3.19 | 0.1 | 15.45 | 112 |
| CAN | 1980 | 11 | 7.92 | 1.51 | 5.8 | 12 | 37 |
| CAN | 1981 | 2 | 3.13 | 2.07 | -0.67 | 8.2 | 31 |
| CAN | 1981 | 3 | 5.22 | 2.57 | 1.2 | 9.44 | 86 |
| CAN | 1981 | 4 | 5.73 | 1.49 | 4.6 | 8.3 | 10 |
| CAN | 1981 | 5 | 5.40 | 0.87 | 3.7 | 7.4 | 33 |
| CAN | 1981 | 7 | 6.31 | 2.30 | 1.78 | 12.27 | 145 |
| CAN | 1981 | 9 | 8.95 | 0.00 | 8.95 | 8.95 | 2 |
| CAN | 1981 | 10 | 7.66 | 2.50 | 2.7 | 14.77 | 134 |
| CAN | 1981 | 11 | 7.64 | 1.16 | 5.6 | 9.8 | 40 |
| CAN | 1982 | 3 | 3.79 | 2.86 | -1 | 9.31 | 126 |
| CAN | 1982 | 4 | 6.37 | 2.09 | 2.9 | 9.2 | 11 |
| CAN | 1982 | 5 | 5.74 | 1.35 | 3.7 | 8.7 | 28 |
| CAN | 1982 | 7 | 5.33 | 2.78 | 0.67 | 12.6 | 150 |
| CAN | 1982 | 9 | 6.74 | 4.50 | 1.1 | 15.42 | 29 |
| CAN | 1982 | 10 | 6.20 | 3.00 | 1.14 | 14.72 | 137 |
| CAN | 1982 | 11 | 8.31 | 1.29 | 6.2 | 10.3 | 27 |
| CAN | 1983 | 3 | 3.72 | 2.36 | 0.65 | 9.22 | 68 |
| CAN | 1983 | 4 | 5.94 | 2.39 | 1.52 | 10.89 | 107 |
| CAN | 1983 | 7 | 5.56 | 2.50 | 1.3 | 10.6 | 141 |
| CAN | 1983 | 10 | 6.30 | 3.10 | 1.6 | 16.7 | 165 |
| CAN | 1983 | 11 | 8.60 | 1.13 | 6.4 | 10.7 | 35 |
| CAN | 1984 | 3 | 5.46 | 2.87 | -0.12 | 12.15 | 141 |
| CAN | 1984 | 4 | 5.34 | 1.47 | 3.1 | 8.1 | 40 |
| CAN | 1984 | 7 | 7.45 | 2.33 | 2.44 | 13.64 | 130 |
| CAN | 1984 | 8 | 3.89 | 2.37 | 1.58 | 8.41 | 13 |
| CAN | 1984 | 10 | 9.15 | 2.43 | 2.8 | 12.4 | 109 |
| CAN | 1984 | 11 | 9.37 | 1.65 | 6.6 | 11.3 | 10 |
| CAN | 1985 | 3 | 6.79 | 3.47 | -0.78 | 12.94 | 46 |
| CAN | 1985 | 4 | 5.27 | 1.81 | 2.7 | 7.8 | 11 |
| CAN | 1985 | 7 | 5.53 | 3.10 | -0.3 | 11.89 | 115 |
| CAN | 1985 | 11 | 8.75 | 1.52 | 6.5 | 11.6 | 14 |
| CAN | 1986 | 3 | 4.27 | 3.33 | -0.06 | 13.41 | 113 |
| CAN | 1986 | 4 | 7.08 | 1.79 | 5 | 10.1 | 16 |
| CAN | 1986 | 7 | 6.23 | 2.89 | 0.03 | 11.29 | 169 |
| CAN | 1986 | 10 | 8.93 | 1.65 | 3.61 | 11.84 | 103 |
| CAN | 1986 | 11 | 8.51 | 1.19 | 6.7 | 10.3 | 18 |
| CAN | 1987 | 3 | 2.72 | 2.73 | -0.57 | 10.46 | 106 |
| CAN | 1987 | 4 | 5.08 | 2.29 | 0.33 | 9.02 | 24 |
| CAN | 1987 | 6 | 4.81 | 2.41 | 2.68 | 9.31 | 9 |
| CAN | 1987 | 7 | 4.84 | 2.85 | -0.32 | 10.12 | 158 |
| CAN | 1987 | 8 | 5.83 | 3.72 | 1.36 | 14.36 | 17 |
| CAN | 1987 | 10 | 7.70 | 1.40 | 5.7 | 10.8 | 17 |
| CAN | 1988 | 3 | 4.50 | 2.78 | 0.08 | 10.51 | 119 |
| CAN | 1988 | 4 | 5.61 | 1.55 | 3.1 | 7.8 | 21 |
| CAN | 1988 | 7 | 5.67 | 2.51 | 0.97 | 13.13 | 176 |
| CAN | 1988 | 10 | 8.07 | 1.19 | 6.4 | 9.9 | 18 |
| CAN | 1989 | 2 | 5.70 | 1.32 | 4.24 | 8.73 | 35 |
| CAN | 1989 | 3 | 2.65 | 2.85 | -1.53 | 9.81 | 83 |
| CAN | 1989 | 4 | 6.01 | 1.34 | 4 | 8.1 | 8 |
| CAN | 1989 | 7 | 5.45 | 2.82 | 0.11 | 10.53 | 179 |
| CAN | 1989 | 10 | 7.51 | 1.28 | 5.7 | 10.2 | 11 |
| CAN | 1989 | 11 | 7.28 | 1.48 | 6.3 | 9.9 | 5 |
| CAN | 1990 | 2 | 5.25 | 0.97 | 4.16 | 8.47 | 27 |
| CAN | 1990 | 3 | 2.94 | 3.21 | -1.3 | 12.06 | 83 |
| CAN | 1990 | 4 | 5.31 | 1.49 | 2.8 | 7.4 | 13 |
| CAN | 1990 | 7 | 5.19 | 3.01 | -0.11 | 11.31 | 229 |
| CAN | 1990 | 10 | 7.75 | 1.37 | 6 | 10.3 | 18 |
| CAN | 1991 | 2 | 5.78 | 0.98 | 5.01 | 9.75 | 43 |
| CAN | 1991 | 3 | 3.20 | 3.27 | -0.74 | 11.74 | 74 |
| CAN | 1991 | 4 | 5.94 | 1.61 | 2.6 | 8 | 31 |
| CAN | 1991 | 7 | 5.04 | 2.87 | 0.28 | 13.56 | 252 |
| CAN | 1991 | 10 | 8.69 | 1.48 | 6.1 | 10.9 | 32 |
| CAN | 1992 | 2 | 4.68 | 0.48 | 4.19 | 6.62 | 35 |
| CAN | 1992 | 3 | 2.85 | 2.45 | -0.98 | 8.36 | 68 |
| CAN | 1992 | 4 | 6.31 | 1.64 | 2.8 | 8.1 | 24 |
| CAN | 1992 | 6 | 6.06 | 1.89 | 2.06 | 9.15 | 54 |
| CAN | 1992 | 7 | 4.43 | 3.17 | -0.62 | 11.18 | 141 |
| CAN | 1992 | 10 | 7.58 | 1.25 | 5.1 | 9.4 | 27 |
| CAN | 1993 | 3 | 3.65 | 2.22 | -0.59 | 9.68 | 77 |
| CAN | 1993 | 4 | 5.21 | 1.90 | 2.4 | 8 | 29 |
| CAN | 1993 | 7 | 5.75 | 2.70 | 0.27 | 10.71 | 197 |
| CAN | 1993 | 8 | 3.19 | 1.36 | 1.12 | 4.6 | 5 |
| CAN | 1993 | 10 | 8.17 | 1.58 | 5 | 10.6 | 32 |
| CAN | 1994 | 2 | 4.50 | 2.38 | 0.59 | 11.56 | 43 |
| CAN | 1994 | 3 | 5.77 | 4.03 | -1.36 | 11.6 | 60 |
| CAN | 1994 | 4 | 6.88 | 2.15 | 3.4 | 10.8 | 24 |
| CAN | 1994 | 7 | 6.06 | 3.24 | 0.53 | 13.16 | 186 |
| CAN | 1994 | 8 | 8.71 | 0.90 | 6.8 | 10.2 | 12 |
| CAN | 1994 | 10 | 9.55 | 0.96 | 7 | 11.1 | 26 |
| CAN | 1995 | 2 | 5.04 | 1.66 | 1.43 | 9.3 | 41 |
| CAN | 1995 | 3 | 3.05 | 2.81 | -0.59 | 11.06 | 95 |
| CAN | 1995 | 4 | 6.27 | 1.73 | 3.6 | 8.8 | 29 |
| CAN | 1995 | 6 | 7.17 | 2.28 | 2.05 | 10.27 | 45 |
| CAN | 1995 | 7 | 4.95 | 3.11 | 0.27 | 11.86 | 149 |
| CAN | 1995 | 8 | 7.78 | 1.38 | 5.6 | 9.7 | 16 |
| CAN | 1995 | 10 | 8.54 | 1.40 | 6 | 11.2 | 38 |
| CAN | 1996 | 2 | 5.60 | 1.15 | 4.34 | 8.75 | 44 |
| CAN | 1996 | 3 | 3.67 | 2.70 | 0.78 | 9.07 | 41 |
| CAN | 1996 | 4 | 6.27 | 1.70 | 3.3 | 9.3 | 22 |
| CAN | 1996 | 7 | 5.18 | 2.68 | 0.53 | 11.62 | 189 |
| CAN | 1996 | 10 | 8.01 | 0.82 | 7.1 | 10.5 | 27 |
| CAN | 1997 | 2 | 5.36 | 1.24 | 4.57 | 8.91 | 13 |
| CAN | 1997 | 3 | 4.39 | 3.22 | 0.06 | 10.45 | 87 |
| CAN | 1997 | 4 | 5.96 | 1.56 | 3 | 8.3 | 31 |
| CAN | 1997 | 7 | 5.50 | 2.70 | 0.93 | 11.91 | 198 |
| CAN | 1997 | 10 | 8.38 | 1.09 | 6.5 | 10.3 | 28 |
| CAN | 1998 | 2 | 5.07 | 0.99 | 3.42 | 7.58 | 13 |
| CAN | 1998 | 4 | 6.02 | 1.24 | 3.7 | 7.9 | 36 |
| CAN | 1998 | 7 | 4.67 | 2.21 | 0.39 | 9.69 | 170 |
| CAN | 1998 | 10 | 7.29 | 0.97 | 6.1 | 9.2 | 26 |
| CAN | 1998 | 11 | 6.83 | 0.61 | 6 | 8.1 | 15 |
| CAN | 1999 | 2 | 6.85 | 2.16 | 4.79 | 11.51 | 15 |
| CAN | 1999 | 3 | 4.35 | 3.06 | -0.15 | 10.89 | 89 |
| CAN | 1999 | 4 | 5.88 | 1.41 | 3.3 | 8.3 | 26 |
| CAN | 1999 | 7 | 6.28 | 2.59 | 1.93 | 11.47 | 190 |
| CAN | 1999 | 10 | 10.80 | 1.17 | 8.5 | 12.5 | 16 |
| CAN | 1999 | 11 | 8.22 | 1.53 | 6.1 | 10.8 | 17 |
| CAN | 2000 | 2 | 6.84 | 1.52 | 5.16 | 9.33 | 15 |
| CAN | 2000 | 3 | 5.17 | 2.80 | 1.22 | 12.96 | 104 |
| CAN | 2000 | 4 | 6.92 | 1.17 | 4.8 | 8.7 | 21 |
| CAN | 2000 | 5 | 5.71 | 0.42 | 5.1 | 6.4 | 9 |
| CAN | 2000 | 7 | 6.35 | 2.51 | 1.69 | 11.14 | 209 |
| CAN | 2000 | 10 | 8.59 | 1.08 | 6.9 | 10.2 | 27 |
| CAN | 2001 | 2 | 5.31 | 2.10 | -0.01 | 10.71 | 34 |
| CAN | 2001 | 3 | 3.86 | 3.11 | -0.16 | 10.4 | 71 |
| CAN | 2001 | 4 | 6.34 | 1.83 | 3.56 | 8.61 | 29 |
| CAN | 2001 | 7 | 4.99 | 2.68 | 0.71 | 11.85 | 202 |
| CAN | 2001 | 10 | 8.40 | 1.04 | 6.87 | 10.77 | 25 |
| CAN | 2002 | 2 | 5.99 | 0.74 | 5.16 | 8.03 | 23 |
| CAN | 2002 | 3 | 3.70 | 2.42 | 0.52 | 9.41 | 107 |
| CAN | 2002 | 4 | 6.52 | 1.20 | 4.37 | 8.05 | 28 |
| CAN | 2002 | 7 | 6.17 | 2.93 | 1.1 | 12.02 | 200 |
| CAN | 2002 | 10 | 9.49 | 1.50 | 7.25 | 11.59 | 27 |
| CAN | 2003 | 2 | 5.63 | 1.80 | 2.85 | 8.36 | 14 |
| CAN | 2003 | 3 | 3.50 | 3.57 | -1.44 | 10.57 | 99 |
| CAN | 2003 | 4 | 5.49 | 1.94 | 2.3 | 8.68 | 28 |
| CAN | 2003 | 7 | 5.16 | 3.09 | -0.06 | 10.64 | 205 |
| CAN | 2003 | 10 | 8.24 | 1.27 | 5.95 | 10.25 | 26 |
| CAN | 2004 | 2 | 4.58 | 1.56 | 3.44 | 8.71 | 14 |
| CAN | 2004 | 4 | 4.87 | 1.88 | 2.18 | 8.06 | 26 |
| CAN | 2004 | 7 | 4.17 | 2.51 | 0.82 | 9.68 | 184 |
| CAN | 2004 | 10 | 6.78 | 1.30 | 4.55 | 9.36 | 23 |
| CAN | 2005 | 2 | 4.40 | NA | 4.4 | 4.4 | 1 |
| CAN | 2005 | 3 | 4.96 | 2.27 | 3.28 | 7.54 | 3 |
| CAN | 2005 | 4 | 5.48 | 1.30 | 3 | 7.78 | 29 |
| CAN | 2005 | 6 | 6.19 | 1.74 | 1.95 | 8.87 | 21 |
| CAN | 2005 | 7 | 8.06 | 1.26 | 6.22 | 9.49 | 9 |
| CAN | 2005 | 10 | 8.62 | 1.16 | 7.1 | 10.88 | 26 |
| CAN | 2005 | 11 | 6.50 | NA | 6.5 | 6.5 | 1 |
| CAN | 2006 | 3 | 6.15 | NA | 6.15 | 6.15 | 1 |
| CAN | 2006 | 4 | 6.68 | 1.47 | 4.07 | 9.14 | 29 |
| CAN | 2006 | 7 | 6.57 | 2.68 | 1.37 | 13.19 | 197 |
| CAN | 2006 | 8 | 6.80 | 2.20 | 3.46 | 10.34 | 8 |
| CAN | 2006 | 10 | 9.15 | 1.24 | 7.09 | 11.62 | 28 |
| CAN | 2007 | 2 | 4.99 | 1.01 | 3.96 | 7.16 | 20 |
| CAN | 2007 | 3 | 2.70 | 1.99 | -0.7 | 7.5 | 66 |
| CAN | 2007 | 4 | 5.27 | 1.39 | 3.04 | 7.11 | 29 |
| CAN | 2007 | 7 | 4.99 | 2.39 | 0.61 | 10.17 | 156 |
| CAN | 2007 | 8 | 4.78 | 3.38 | 1.97 | 10.31 | 6 |
| CAN | 2007 | 10 | 7.26 | 0.81 | 5.95 | 9.41 | 28 |
| CAN | 2008 | 3 | 3.64 | 2.39 | -0.39 | 9.59 | 112 |
| CAN | 2008 | 4 | 6.38 | 0.66 | 5.43 | 7.1 | 8 |
| CAN | 2008 | 5 | 5.20 | NA | 5.2 | 5.2 | 1 |
| CAN | 2008 | 7 | 5.39 | 2.54 | 0.6 | 11.44 | 157 |
| CAN | 2008 | 10 | 9.01 | 1.18 | 7.44 | 10.08 | 6 |
| CAN | 2009 | 4 | 6.04 | 1.22 | 3.47 | 7.27 | 9 |
| CAN | 2009 | 5 | 5.69 | 1.07 | 3.71 | 7.34 | 34 |
| CAN | 2009 | 7 | 5.87 | 2.71 | 0.62 | 10.91 | 196 |
| CAN | 2009 | 11 | 8.55 | 1.23 | 5.92 | 10.13 | 28 |
| CAN | 2010 | 4 | 6.60 | 1.32 | 4.2 | 8.77 | 39 |
| CAN | 2010 | 7 | 6.05 | 2.97 | 0.36 | 11.66 | 172 |
| CAN | 2010 | 8 | 7.98 | 2.27 | 3.56 | 11.36 | 10 |
| CAN | 2010 | 11 | 9.89 | 1.00 | 8.12 | 11.28 | 28 |
| CAN | 2010 | 12 | 8.35 | 0.03 | 8.32 | 8.38 | 3 |
| CAN | 2011 | 4 | 7.68 | 1.47 | 4.67 | 9.3 | 17 |
| CAN | 2011 | 5 | 7.20 | 1.34 | 4.75 | 8.87 | 16 |
| CAN | 2011 | 7 | 6.58 | 2.65 | 2.06 | 11.08 | 188 |
| CAN | 2011 | 8 | 5.04 | 1.97 | 2.19 | 10.98 | 57 |
| CAN | 2011 | 10 | 9.15 | 0.53 | 8.38 | 9.6 | 4 |
| CAN | 2011 | 11 | 8.70 | 0.68 | 8.03 | 9.85 | 15 |
| CAN | 2012 | 4 | 7.37 | 1.20 | 5.36 | 8.53 | 24 |
| CAN | 2012 | 5 | 7.56 | 0.74 | 6.54 | 8.49 | 10 |
| USA | 1963 | 7 | 6.90 | 1.54 | 4.7 | 12.5 | 47 |
| USA | 1963 | 8 | 10.29 | 2.39 | 5.3 | 15 | 77 |
| USA | 1963 | 11 | 7.06 | 1.23 | 5.7 | 9.9 | 12 |
| USA | 1963 | 12 | 9.29 | 3.09 | 4.8 | 14.4 | 39 |
| USA | 1964 | 1 | 5.27 | 0.94 | 2.3 | 7.2 | 50 |
| USA | 1964 | 2 | 5.58 | 1.90 | 1.1 | 10.8 | 89 |
| USA | 1964 | 7 | 7.70 | 1.96 | 4.5 | 13.5 | 37 |
| USA | 1964 | 8 | 7.62 | 2.88 | 4 | 15 | 91 |
| USA | 1964 | 10 | 9.69 | 1.80 | 5.7 | 13.3 | 37 |
| USA | 1964 | 11 | 6.62 | 1.33 | 4.7 | 10.8 | 41 |
| USA | 1964 | 12 | 6.32 | 1.22 | 4.9 | 7.8 | 9 |
| USA | 1965 | 2 | 3.89 | 1.23 | 1.4 | 5.7 | 44 |
| USA | 1965 | 3 | 3.47 | 0.68 | 2.2 | 5 | 19 |
| USA | 1965 | 4 | 5.14 | 2.43 | 2 | 10.5 | 65 |
| USA | 1965 | 7 | 6.93 | 2.60 | 3 | 13.9 | 62 |
| USA | 1965 | 8 | 7.97 | 2.39 | 2.5 | 13 | 68 |
| USA | 1965 | 10 | 7.36 | 2.15 | 4 | 13.5 | 63 |
| USA | 1965 | 11 | 10.81 | 1.18 | 8.5 | 13 | 34 |
| USA | 1966 | 1 | 4.87 | 1.16 | 2.5 | 6.2 | 20 |
| USA | 1966 | 2 | 4.57 | 1.61 | 1.2 | 9 | 120 |
| USA | 1966 | 10 | 6.84 | 2.66 | 4 | 13.2 | 38 |
| USA | 1966 | 11 | 9.46 | 1.76 | 5.1 | 13.2 | 69 |
| USA | 1967 | 10 | 10.77 | 2.50 | 7.2 | 17.8 | 56 |
| USA | 1967 | 11 | 6.99 | 1.82 | 4 | 10.6 | 31 |
| USA | 1967 | 12 | 7.78 | 2.04 | 0 | 11.3 | 40 |
| USA | 1968 | 3 | 6.63 | 3.13 | 1 | 11.4 | 95 |
| USA | 1968 | 4 | 4.75 | 0.84 | 3.3 | 6.4 | 38 |
| USA | 1968 | 5 | 6.55 | 0.07 | 6.5 | 6.6 | 2 |
| USA | 1968 | 10 | 11.88 | 2.89 | 6.9 | 20.7 | 82 |
| USA | 1968 | 11 | 8.91 | 2.74 | 4.5 | 13.5 | 49 |
| USA | 1969 | 3 | 6.50 | 2.75 | 2.1 | 13.3 | 131 |
| USA | 1969 | 4 | 5.14 | 1.29 | 3 | 7.6 | 21 |
| USA | 1969 | 7 | 9.15 | 2.30 | 4.9 | 14.2 | 85 |
| USA | 1969 | 8 | 8.86 | 2.80 | 1.1 | 15.5 | 94 |
| USA | 1969 | 10 | 12.33 | 2.80 | 8.1 | 20.7 | 65 |
| USA | 1969 | 11 | 7.81 | 1.85 | 5.1 | 13.2 | 41 |
| USA | 1970 | 3 | 5.14 | 1.81 | 3.1 | 11.9 | 59 |
| USA | 1970 | 4 | 8.39 | 3.03 | 3.8 | 15.8 | 95 |
| USA | 1970 | 9 | 11.23 | 3.72 | 5.1 | 18.3 | 17 |
| USA | 1970 | 10 | 10.27 | 2.30 | 5.7 | 14.6 | 81 |
| USA | 1970 | 11 | 7.94 | 1.61 | 4.2 | 10.9 | 29 |
| USA | 1971 | 3 | 5.84 | 3.02 | 2.2 | 12.3 | 77 |
| USA | 1971 | 4 | 7.36 | 2.49 | 3.2 | 14.3 | 53 |
| USA | 1971 | 9 | 10.05 | 2.14 | 7.2 | 13.8 | 8 |
| USA | 1971 | 10 | 10.94 | 2.84 | 6.2 | 16.6 | 132 |
| USA | 1971 | 11 | 7.90 | 0.92 | 6 | 10.2 | 21 |
| USA | 1972 | 2 | 5.61 | 1.27 | 4.2 | 8 | 40 |
| USA | 1972 | 3 | 8.12 | 3.00 | 3 | 16.1 | 109 |
| USA | 1972 | 4 | 5.63 | 1.97 | 3.2 | 13 | 58 |
| USA | 1972 | 7 | 4.70 | NA | 4.7 | 4.7 | 1 |
| USA | 1972 | 9 | 11.37 | 1.95 | 8 | 17.2 | 23 |
| USA | 1972 | 10 | 12.11 | 3.33 | 5.8 | 20.8 | 174 |
| USA | 1972 | 11 | 13.02 | 2.67 | 5.5 | 20.9 | 168 |
| USA | 1972 | 12 | 15.05 | 1.81 | 11.5 | 18 | 19 |
| USA | 1973 | 3 | 8.08 | 3.10 | 2.9 | 15.5 | 106 |
| USA | 1973 | 4 | 6.56 | 2.62 | 3.5 | 13.6 | 93 |
| USA | 1973 | 5 | 10.88 | 2.61 | 5.6 | 20.1 | 154 |
| USA | 1973 | 6 | 9.74 | 2.20 | 6.1 | 16 | 37 |
| USA | 1973 | 9 | 14.31 | 3.61 | 9.9 | 20.8 | 35 |
| USA | 1973 | 10 | 14.68 | 3.85 | 5.7 | 20.9 | 231 |
| USA | 1973 | 11 | 10.15 | 2.75 | 6.3 | 14.3 | 46 |
| USA | 1974 | 3 | 10.47 | 2.75 | 3.2 | 15.3 | 54 |
| USA | 1974 | 4 | 9.06 | 3.40 | 4 | 17.3 | 246 |
| USA | 1974 | 5 | 8.37 | 0.08 | 8.3 | 8.5 | 6 |
| USA | 1974 | 9 | 13.94 | 3.20 | 7.9 | 20.8 | 79 |
| USA | 1974 | 10 | 12.70 | 4.12 | 5.8 | 20.9 | 178 |
| USA | 1974 | 11 | 8.50 | 2.00 | 5 | 10.1 | 7 |
| USA | 1975 | 3 | 7.15 | 2.61 | 3.3 | 12.5 | 113 |
| USA | 1975 | 4 | 6.49 | 2.02 | 3 | 12.3 | 77 |
| USA | 1975 | 5 | 5.98 | 1.11 | 4.5 | 7.8 | 18 |
| USA | 1975 | 10 | 11.88 | 3.38 | 5.8 | 18.4 | 246 |
| USA | 1975 | 11 | 14.70 | 3.54 | 6.5 | 19 | 72 |
| USA | 1976 | 3 | 8.01 | 2.83 | 3.8 | 16.1 | 218 |
| USA | 1976 | 4 | 6.61 | 1.67 | 2.2 | 9.9 | 90 |
| USA | 1976 | 5 | 4.10 | NA | 4.1 | 4.1 | 1 |
| USA | 1976 | 9 | 11.99 | 1.93 | 8.6 | 16.3 | 22 |
| USA | 1976 | 10 | 14.22 | 3.57 | 7.2 | 20.9 | 217 |
| USA | 1976 | 11 | 8.95 | 1.54 | 6.9 | 14.8 | 43 |
| USA | 1977 | 3 | 7.38 | 2.75 | 1.9 | 15.6 | 79 |
| USA | 1977 | 4 | 6.01 | 1.95 | 3.3 | 11.3 | 173 |
| USA | 1977 | 5 | 5.23 | 1.04 | 3.8 | 7.7 | 43 |
| USA | 1977 | 7 | 12.59 | 2.85 | 9 | 20.8 | 50 |
| USA | 1977 | 8 | 10.77 | 4.03 | 4.9 | 20.6 | 197 |
| USA | 1977 | 9 | 13.01 | 2.56 | 9.5 | 20.1 | 46 |
| USA | 1977 | 10 | 13.41 | 3.51 | 5.2 | 20.6 | 216 |
| USA | 1977 | 11 | 8.97 | 2.04 | 5.4 | 12.9 | 39 |
| USA | 1977 | 12 | 7.10 | 1.54 | 5.5 | 9.7 | 23 |
| USA | 1978 | 1 | 3.13 | 0.78 | 1.6 | 4.9 | 78 |
| USA | 1978 | 3 | 7.08 | 2.95 | 3.7 | 15.9 | 86 |
| USA | 1978 | 4 | 4.95 | 1.57 | 2.3 | 10.8 | 188 |
| USA | 1978 | 5 | 6.18 | 1.76 | 3.2 | 9.3 | 29 |
| USA | 1978 | 7 | 13.67 | 5.38 | 4.7 | 20.9 | 29 |
| USA | 1978 | 8 | 11.08 | 4.08 | 4.9 | 20.4 | 223 |
| USA | 1978 | 9 | 13.41 | 4.37 | 6.5 | 20.8 | 160 |
| USA | 1978 | 10 | 9.57 | 3.24 | 4.9 | 15.1 | 159 |
| USA | 1978 | 11 | 10.32 | 2.59 | 5.5 | 15.8 | 78 |
| USA | 1979 | 3 | 10.79 | 4.41 | 4.3 | 18.9 | 82 |
| USA | 1979 | 4 | 6.00 | 2.35 | 2.7 | 12 | 287 |
| USA | 1979 | 5 | 6.04 | 1.17 | 3.8 | 7.7 | 38 |
| USA | 1979 | 7 | 11.75 | 4.36 | 6.1 | 19.2 | 33 |
| USA | 1979 | 8 | 11.24 | 4.20 | 4.6 | 20.6 | 210 |
| USA | 1979 | 9 | 14.53 | 4.21 | 7.5 | 20.8 | 59 |
| USA | 1979 | 10 | 12.73 | 2.83 | 5.2 | 19.1 | 269 |
| USA | 1979 | 11 | 7.70 | 2.02 | 4.9 | 15.1 | 103 |
| USA | 1980 | 3 | 10.72 | 4.11 | 4.8 | 20.1 | 139 |
| USA | 1980 | 4 | 6.25 | 2.32 | 3 | 12.7 | 273 |
| USA | 1980 | 5 | 5.79 | 1.17 | 4.8 | 9.7 | 17 |
| USA | 1980 | 7 | 11.14 | 3.85 | 4.9 | 20.2 | 117 |
| USA | 1980 | 8 | 9.26 | 2.92 | 4.9 | 16.3 | 92 |
| USA | 1980 | 9 | 14.13 | 4.68 | 7.3 | 20.9 | 55 |
| USA | 1980 | 10 | 12.34 | 2.76 | 6.5 | 19.3 | 183 |
| USA | 1980 | 11 | 7.34 | 1.50 | 5.1 | 9.4 | 35 |
| USA | 1981 | 1 | 6.42 | 3.51 | -1.4 | 12.8 | 86 |
| USA | 1981 | 3 | 10.11 | 3.84 | 4.9 | 17.7 | 122 |
| USA | 1981 | 4 | 6.50 | 2.28 | 3.3 | 12.3 | 167 |
| USA | 1981 | 5 | 5.61 | 1.24 | 3.7 | 9.9 | 61 |
| USA | 1981 | 6 | 11.16 | 3.13 | 5.6 | 16.8 | 42 |
| USA | 1981 | 7 | 9.70 | 3.25 | 4.8 | 16.6 | 121 |
| USA | 1981 | 9 | 14.31 | 4.41 | 6.6 | 20.8 | 78 |
| USA | 1981 | 10 | 11.07 | 2.81 | 5.3 | 17.2 | 159 |
| USA | 1981 | 11 | 7.44 | 2.03 | 5 | 10.8 | 31 |
| USA | 1981 | 12 | 10.80 | NA | 10.8 | 10.8 | 1 |
| USA | 1982 | 3 | 11.70 | 4.77 | 2.53 | 20.1 | 95 |
| USA | 1982 | 4 | 5.86 | 2.25 | 3.1 | 12.1 | 95 |
| USA | 1982 | 5 | 5.10 | 0.90 | 3.3 | 6.8 | 32 |
| USA | 1982 | 9 | 13.73 | 4.65 | 6.5 | 20.8 | 51 |
| USA | 1982 | 10 | 12.01 | 3.33 | 5.2 | 18.7 | 154 |
| USA | 1982 | 11 | 8.23 | 2.16 | 6.1 | 12.1 | 23 |
| USA | 1983 | 3 | 9.14 | 4.26 | 4.4 | 19.4 | 168 |
| USA | 1983 | 4 | 6.41 | 2.10 | 4.1 | 12.4 | 125 |
| USA | 1983 | 9 | 13.66 | 4.06 | 5.3 | 20.8 | 78 |
| USA | 1983 | 10 | 12.07 | 3.38 | 5.6 | 19.3 | 137 |
| USA | 1983 | 11 | 7.71 | 1.06 | 5.5 | 9.9 | 25 |
| USA | 1984 | 3 | 8.27 | 4.43 | 2.9 | 20.4 | 214 |
| USA | 1984 | 4 | 5.82 | 1.57 | 3.2 | 11.3 | 77 |
| USA | 1984 | 9 | 14.36 | 4.82 | 6.8 | 20.9 | 110 |
| USA | 1984 | 10 | 11.16 | 3.06 | 6.2 | 16.2 | 49 |
| USA | 1984 | 11 | 8.28 | 0.25 | 8 | 8.6 | 4 |
| USA | 1985 | 2 | 10.20 | 2.03 | 7 | 12.9 | 6 |
| USA | 1985 | 3 | 7.71 | 3.87 | 3 | 19.7 | 58 |
| USA | 1985 | 4 | 5.84 | 1.20 | 4.3 | 9.1 | 19 |
| USA | 1985 | 9 | 12.24 | 2.46 | 9.7 | 16.8 | 7 |
| USA | 1985 | 10 | 12.98 | 3.67 | 5.9 | 20.9 | 67 |
| USA | 1985 | 11 | 9.05 | 1.57 | 6.9 | 10.5 | 6 |
| USA | 1986 | 3 | 7.09 | 3.15 | 3.3 | 17.4 | 103 |
| USA | 1986 | 4 | 7.39 | 2.29 | 4.3 | 13.5 | 48 |
| USA | 1986 | 9 | 14.97 | 3.70 | 5.9 | 20.8 | 34 |
| USA | 1986 | 10 | 11.72 | 2.81 | 5.7 | 19.2 | 93 |
| USA | 1986 | 11 | 8.40 | 1.07 | 6.8 | 9.1 | 4 |
| USA | 1987 | 3 | 6.19 | 3.08 | 3.45 | 20 | 109 |
| USA | 1987 | 4 | 6.81 | 2.53 | 3.5 | 12.2 | 86 |
| USA | 1987 | 9 | 13.48 | 4.24 | 6.6 | 20.7 | 45 |
| USA | 1987 | 10 | 9.73 | 3.13 | 4.9 | 15.8 | 56 |
| USA | 1988 | 3 | 6.08 | 2.45 | 3.2 | 11.8 | 192 |
| USA | 1988 | 4 | 5.63 | 1.60 | 4 | 8.4 | 14 |
| USA | 1988 | 9 | 12.66 | 3.77 | 7.1 | 20.7 | 64 |
| USA | 1988 | 10 | 8.83 | 2.69 | 5.5 | 15.3 | 42 |
| USA | 1989 | 2 | 5.76 | 2.45 | 4.08 | 11.82 | 16 |
| USA | 1989 | 3 | 6.21 | 2.67 | 1.9 | 13.5 | 117 |
| USA | 1989 | 4 | 6.42 | 1.24 | 5.1 | 8.1 | 6 |
| USA | 1989 | 7 | 7.21 | 0.35 | 6.76 | 7.6 | 4 |
| USA | 1989 | 9 | 13.34 | 4.01 | 7.1 | 20.3 | 36 |
| USA | 1989 | 10 | 10.47 | 3.53 | 5.1 | 17.7 | 63 |
| USA | 1990 | 2 | 5.55 | 1.83 | 4.17 | 11.81 | 37 |
| USA | 1990 | 3 | 7.04 | 3.28 | 3.73 | 20.6 | 116 |
| USA | 1990 | 4 | 5.71 | 1.30 | 3.3 | 7.8 | 17 |
| USA | 1990 | 9 | 13.81 | 3.92 | 7.7 | 20.9 | 45 |
| USA | 1990 | 10 | 5.98 | 0.67 | 5.4 | 6.9 | 4 |
| USA | 1991 | 2 | 6.00 | 1.66 | 3.96 | 13.67 | 92 |
| USA | 1991 | 3 | 8.18 | 3.75 | 4.3 | 20 | 180 |
| USA | 1991 | 4 | 5.74 | 1.15 | 3.7 | 8.4 | 65 |
| USA | 1991 | 7 | 5.05 | 0.21 | 4.9 | 5.2 | 2 |
| USA | 1991 | 9 | 14.21 | 3.83 | 7.5 | 20.8 | 116 |
| USA | 1991 | 10 | 10.29 | 3.06 | 5.5 | 16.8 | 103 |
| USA | 1992 | 2 | 6.94 | 2.77 | 2.1 | 13.8 | 84 |
| USA | 1992 | 3 | 6.39 | 3.36 | 2.5 | 20.6 | 263 |
| USA | 1992 | 4 | 5.63 | 1.69 | 3.3 | 8.3 | 60 |
| USA | 1992 | 9 | 14.27 | 4.29 | 6.8 | 19.9 | 90 |
| USA | 1992 | 10 | 10.58 | 3.14 | 4.9 | 17.2 | 113 |
| USA | 1993 | 2 | 6.99 | 2.49 | 1.4 | 12.3 | 106 |
| USA | 1993 | 3 | 5.80 | 3.43 | 2.3 | 17.8 | 175 |
| USA | 1993 | 4 | 5.19 | 2.20 | 2.2 | 12.9 | 100 |
| USA | 1993 | 7 | 6.77 | 1.85 | 4.2 | 9.3 | 19 |
| USA | 1993 | 8 | 4.88 | 1.01 | 3.4 | 6.6 | 15 |
| USA | 1993 | 9 | 13.18 | 3.47 | 5.4 | 20.9 | 168 |
| USA | 1993 | 10 | 9.72 | 3.40 | 4.9 | 16.5 | 74 |
| USA | 1994 | 2 | 6.66 | 3.33 | 0.3 | 14 | 89 |
| USA | 1994 | 3 | 5.94 | 4.16 | 1.3 | 19.9 | 150 |
| USA | 1994 | 4 | 6.79 | 2.77 | 3 | 13.4 | 98 |
| USA | 1994 | 7 | 6.55 | 1.49 | 5.5 | 8.7 | 4 |
| USA | 1994 | 9 | 15.57 | 3.94 | 7 | 20.9 | 127 |
| USA | 1994 | 10 | 11.53 | 2.82 | 5.8 | 16.2 | 109 |
| USA | 1995 | 2 | 7.22 | 2.70 | 2.2 | 13.87 | 153 |
| USA | 1995 | 3 | 7.57 | 2.97 | 3.8 | 20.4 | 146 |
| USA | 1995 | 4 | 6.73 | 2.60 | 3.6 | 13.4 | 113 |
| USA | 1995 | 8 | 8.91 | 4.27 | 6 | 20.5 | 10 |
| USA | 1995 | 9 | 14.36 | 4.11 | 5.9 | 20.9 | 110 |
| USA | 1995 | 10 | 11.32 | 3.86 | 6 | 19.5 | 75 |
| USA | 1996 | 2 | 6.49 | 2.71 | 1.5 | 12.5 | 154 |
| USA | 1996 | 3 | 5.88 | 2.73 | 3 | 12 | 131 |
| USA | 1996 | 4 | 5.79 | 2.24 | 2.7 | 14.2 | 134 |
| USA | 1996 | 9 | 12.83 | 3.97 | 6.9 | 20.6 | 121 |
| USA | 1996 | 10 | 8.85 | 2.32 | 6 | 14.8 | 74 |
| USA | 1997 | 2 | 7.91 | 2.69 | 3.9 | 13.7 | 111 |
| USA | 1997 | 3 | 7.88 | 3.66 | 3.2 | 20.9 | 220 |
| USA | 1997 | 4 | 6.16 | 1.87 | 3.7 | 12 | 72 |
| USA | 1997 | 9 | 14.58 | 3.84 | 7.5 | 20.8 | 77 |
| USA | 1997 | 10 | 10.93 | 3.12 | 6.1 | 16.3 | 125 |
| USA | 1998 | 2 | 6.77 | 2.52 | 4.2 | 15.9 | 135 |
| USA | 1998 | 3 | 6.68 | 2.29 | 3.7 | 17.1 | 197 |
| USA | 1998 | 4 | 5.74 | 1.08 | 3.4 | 7.4 | 85 |
| USA | 1998 | 9 | 12.83 | 4.23 | 7.8 | 20.7 | 59 |
| USA | 1998 | 10 | 10.90 | 3.29 | 5.2 | 18.4 | 140 |
| USA | 1998 | 11 | 6.93 | 0.98 | 6 | 9.6 | 12 |
| USA | 1999 | 2 | 8.88 | 3.03 | 3.7 | 14.9 | 142 |
| USA | 1999 | 3 | 8.05 | 3.86 | 3.9 | 20.4 | 165 |
| USA | 1999 | 4 | 6.26 | 1.86 | 3.6 | 12.4 | 99 |
| USA | 1999 | 9 | 15.05 | 3.67 | 9.2 | 20.9 | 44 |
| USA | 1999 | 10 | 13.96 | 3.37 | 5.6 | 18.9 | 139 |
| USA | 1999 | 11 | 7.74 | 1.52 | 6.1 | 12.8 | 28 |
| USA | 2000 | 2 | 8.55 | 3.20 | 2.3 | 15 | 136 |
| USA | 2000 | 3 | 7.81 | 3.11 | 4.4 | 20.4 | 145 |
| USA | 2000 | 4 | 7.46 | 2.03 | 4.6 | 13.3 | 110 |
| USA | 2000 | 5 | 5.20 | 0.26 | 5 | 5.5 | 3 |
| USA | 2000 | 9 | 13.96 | 3.51 | 6.2 | 20.9 | 150 |
| USA | 2000 | 10 | 10.50 | 3.22 | 6.8 | 16.8 | 62 |
| USA | 2001 | 1 | 9.81 | 2.08 | 7.1 | 13.1 | 16 |
| USA | 2001 | 2 | 7.83 | 3.01 | 2.6 | 13.2 | 162 |
| USA | 2001 | 3 | 6.39 | 2.71 | 3.47 | 16.75 | 138 |
| USA | 2001 | 4 | 6.20 | 1.85 | 3.84 | 12.52 | 102 |
| USA | 2001 | 9 | 14.44 | 4.05 | 5.78 | 20.96 | 145 |
| USA | 2001 | 10 | 9.64 | 3.28 | 5.74 | 17.39 | 77 |
| USA | 2002 | 2 | 9.35 | 2.81 | 4.39 | 16.47 | 145 |
| USA | 2002 | 3 | 8.76 | 2.40 | 5.11 | 14.5 | 152 |
| USA | 2002 | 4 | 7.15 | 1.77 | 4.76 | 13.54 | 103 |
| USA | 2002 | 9 | 13.78 | 4.29 | 5.04 | 20.92 | 95 |
| USA | 2002 | 10 | 11.67 | 3.38 | 6.38 | 18.25 | 104 |
| USA | 2003 | 2 | 6.67 | 3.01 | 1.76 | 12.28 | 83 |
| USA | 2003 | 3 | 6.05 | 3.72 | 1.94 | 20.21 | 151 |
| USA | 2003 | 4 | 5.53 | 1.90 | 3.21 | 11.58 | 88 |
| USA | 2003 | 9 | 13.29 | 4.01 | 6.07 | 20.51 | 114 |
| USA | 2003 | 10 | 12.51 | 5.25 | 4.92 | 20.74 | 106 |
| USA | 2004 | 2 | 6.74 | 2.91 | 1.36 | 12.67 | 132 |
| USA | 2004 | 3 | 5.93 | 3.02 | 2.93 | 18.75 | 168 |
| USA | 2004 | 4 | 4.64 | 1.35 | 2.95 | 8.76 | 85 |
| USA | 2004 | 9 | 13.71 | 4.74 | 6.63 | 20.94 | 107 |
| USA | 2004 | 10 | 9.40 | 3.58 | 4.47 | 15.95 | 99 |
| USA | 2005 | 2 | 6.84 | 2.94 | 3.11 | 12.63 | 82 |
| USA | 2005 | 3 | 6.34 | 3.61 | 2.02 | 19.57 | 142 |
| USA | 2005 | 4 | 5.38 | 1.75 | 2.85 | 11.45 | 87 |
| USA | 2005 | 9 | 13.18 | 3.72 | 7.99 | 20.96 | 101 |
| USA | 2005 | 10 | 11.17 | 2.67 | 5.51 | 17.4 | 87 |
| USA | 2005 | 11 | 7.80 | 1.60 | 6.1 | 10.74 | 19 |
| USA | 2006 | 2 | 9.68 | 3.33 | 4.1 | 19.64 | 97 |
| USA | 2006 | 3 | 8.30 | 3.53 | 4.17 | 19.57 | 197 |
| USA | 2006 | 4 | 6.08 | 1.49 | 3.97 | 9.79 | 71 |
| USA | 2006 | 9 | 14.54 | 3.70 | 6.46 | 20.96 | 146 |
| USA | 2006 | 10 | 9.79 | 2.74 | 6.37 | 17.03 | 79 |
| USA | 2007 | 2 | 8.78 | 3.44 | 2.18 | 18.26 | 122 |
| USA | 2007 | 3 | 7.24 | 3.59 | 3.11 | 20.61 | 199 |
| USA | 2007 | 4 | 5.49 | 1.04 | 4.05 | 8.38 | 91 |
| USA | 2007 | 9 | 13.79 | 4.95 | 6.21 | 20.97 | 85 |
| USA | 2007 | 10 | 10.12 | 3.47 | 5.48 | 18.19 | 124 |
| USA | 2008 | 3 | 7.45 | 3.13 | 4.15 | 20.13 | 77 |
| USA | 2008 | 4 | 6.31 | 2.27 | 3.67 | 12.95 | 60 |
| USA | 2008 | 5 | 5.89 | 1.01 | 4.18 | 6.97 | 11 |
| USA | 2008 | 9 | 15.12 | 4.90 | 8.88 | 20.38 | 12 |
| USA | 2008 | 10 | 10.39 | 2.70 | 6.6 | 15.98 | 23 |
| USA | 2009 | 2 | 4.16 | 0.51 | 3.8 | 4.52 | 2 |
| USA | 2009 | 3 | 7.54 | 3.45 | 3.37 | 17.08 | 146 |
| USA | 2009 | 4 | 6.03 | 2.12 | 3.22 | 13.44 | 107 |
| USA | 2009 | 5 | 5.21 | 1.27 | 3.5 | 6.61 | 10 |
| USA | 2009 | 9 | 15.44 | 3.76 | 8.83 | 20.92 | 73 |
| USA | 2009 | 10 | 12.63 | 1.86 | 6.74 | 16.82 | 88 |
| USA | 2009 | 11 | 8.80 | 2.05 | 5.39 | 12.57 | 54 |
| USA | 2010 | 2 | 3.99 | 0.93 | 3.18 | 5.51 | 8 |
| USA | 2010 | 3 | 6.87 | 3.44 | 2.74 | 19.29 | 171 |
| USA | 2010 | 4 | 7.40 | 2.40 | 4.27 | 12.74 | 117 |
| USA | 2010 | 5 | 6.64 | 1.14 | 4.76 | 8.66 | 7 |
| USA | 2010 | 9 | 14.04 | 3.60 | 8.19 | 20.65 | 102 |
| USA | 2010 | 10 | 12.93 | 2.71 | 6.66 | 19.22 | 115 |
| USA | 2010 | 11 | 8.63 | 1.41 | 6.63 | 11.39 | 26 |
| USA | 2010 | 12 | 8.95 | 0.49 | 8.03 | 9.5 | 8 |
| USA | 2011 | 3 | 7.68 | 3.57 | 3.31 | 20.6 | 176 |
| USA | 2011 | 4 | 7.17 | 1.88 | 4.23 | 12 | 80 |
| USA | 2011 | 5 | 6.38 | 1.60 | 4.39 | 8.77 | 26 |
| USA | 2011 | 9 | 14.49 | 3.92 | 8.74 | 20.89 | 137 |
| USA | 2011 | 10 | 12.66 | 3.18 | 7.21 | 18.81 | 99 |
| USA | 2011 | 11 | 9.05 | 1.13 | 7.73 | 11.76 | 25 |
| USA | 2012 | 2 | 11.34 | 1.42 | 9.77 | 12.97 | 5 |
| USA | 2012 | 3 | 9.94 | 2.31 | 6.95 | 16.76 | 140 |
| USA | 2012 | 4 | 7.60 | 1.67 | 5.46 | 13.83 | 107 |
| USA | 2012 | 5 | 7.37 | 0.80 | 6.56 | 8.15 | 3 |
|  |  |  |  |  |  |  |  |
