# Supplementary material for: Thermal Habitat Index of Many Northwest Atlantic Temperate Species Stays Neutral under Warming Projected for 2030 but Changes Radically by 2060
Source: PLoS One. 2014 Mar 5;9(3):e90662. doi: 10.1371/journal.pone.0090662 (PMC3944076; doi:10.1371/journal.pone.0090662)
Supplement: Table S2 — Summary of Species, Functional Groups, Commercial Categories, Name abbreviations. Net change in Realized Thermal Habitat Index in Scenario 2 is also shown. NA refers to not enough probable habitat (insufficient probability estimates >0.5). (DOCX) [file pone.0090662.s003.docx]

**Table S2. Summary of Species, Functional Groups, Commercial Categories, Name abbreviations**. Net change in Realized Thermal Habitat Index in Scenario 2 is also shown. NA refers to not enough probable habitat (insufficient probability estimates >0.5)

| species | Scientific_Name | FxnlGrp | Comm000DollarsCAN | CommercialCAN | GainLossY2030.Canada | Comm000DollarsUS | CommercialUS | GainLossY2030.USA | Name abbreviation in Figures |
| --- | --- | --- | --- | --- | --- | --- | --- | --- | --- |
| Barndoor_Skate | DIPTURUS LAEVIS | Lg.Benthivore | 1.00 | NonComm | 0.00 | 1.00 | NonComm | 2.33 | Brndr_Sk |
| Black Belly_Rosefish | HELICOLENUS DACTYLOPTERUS | Zoopiscivore | 1.00 | NonComm | 11.76 | 0.11 | Low | -7.39 | BlckBl_R |
| Capelin | MALLOTUS VILLOSUS | Planktivore | 1.00 | NonComm | -16.60 | 1.00 | NonComm | NA | Capelin |
| Cod | GADUS MORHUA | Piscivore | 5108.07 | Medium | -3.00 | 15155.51 | High | -7.58 | Cod |
| Cunner | TAUTOGOLABRUS ADSPERSUS | Sm.Benthivore | 1.00 | NonComm | NA | 1.00 | NonComm | 0.00 | Cunner |
| Cusk | BROSME BROSME | Piscivore | 458.28 | Low | 0.50 | 7707.08 | Medium | 13.00 | Cusk |
| Dogfish | SQUALUS ACANTHIAS | Piscivore | 47.95 | Low | 8.00 | 178.61 | Low | 3.99 | Dogfish |
| Dory | ZENOPSIS CONCHIFERA | Sm.Benthivore | 1.00 | NonComm | NA | 1.00 | NonComm | NA | Dory |
| Gulf Stream_flounder | CITHARICHTHYS ARCTIFRONS | Sm.Benthivore | 1.00 | NonComm | NA | 1.00 | NonComm | -0.72 | GlfStrm_ |
| Haddock | MELANOGRAMMUS AEGLEFINUS | Lg.Benthivore | 25949.87 | High | 1.70 | 1060.64 | Low | 1.18 | Haddock |
| Hagfish | MYXINE GLUTINOSA | Lg.Benthivore | 1.00 | NonComm | 17.05 | 1.00 | NonComm | 0.00 | Hagfish |
| Halibut | HIPPOGLOSSUS HIPPOGLOSSUS | Piscivore | 19859.57 | High | 12.73 | 23119.45 | High | NA | Halibut |
| Herring | CLUPEA HARENGUS | Planktivore | 18053.02 | High | -3.75 | 351206.00 | High | -3.37 | Herring |
| Jonah Crab | CANCER BOREALIS | Decapod.Benthivore | 541.66 | Low | 11.29 | 3719.99 | Medium | -6.47 | JonahCrb |
| Little_Skate | LEUCORAJA ERINACEA | Md.Benthivore | 1.00 | NonComm | -5.85 | 9112.18 | High | -2.92 | Littl_Skt |
| Lobster | HOMARUS AMERICANUS | Decapod.Benthivore | 348346.50 | High | 22.40 | 1145.37 | Low | 6.06 | Lobster |
| Longhorn_Sculpin | MYOXOCEPHALUS OCTODECEMSPINOSUS | Sm.Benthivore | 1.00 | NonComm | -3.91 | 1.00 | NonComm | -6.05 | Lnghrn_S |
| Monkfish | LOPHIUS AMERICANUS | Piscivore | 1.00 | NonComm | 10.20 | 17799.02 | High | -5.47 | Monkfish |
| Moustache_Sculpin | TRIGLOPS MURRAYI | Sm.Benthivore | 1.00 | NonComm | -44.20 | 1.00 | NonComm | NA | Mstch_Sc |
| Northern_Shrimp | PANDALUS BOREALIS | Decapod.Benthivore | 54365.81 | High | -4.27 | 30.19 | Low | -2.67 | Nrthrn_S |
| Ocean_Pout | MACROZOARCES AMERICANUS | Lg.Benthivore | 1.00 | NonComm | -11.38 | 0.46 | Low | -6.44 | Ocean_Pt |
| Offshore_Hake | MERLUCCIUS ALBIDUS | Zoopiscivore | 1.00 | NonComm | 0.00 | 1.00 | NonComm | -1.93 | Offshr_H |
| Plaice | HIPPOGLOSSOIDES PLATESSOIDES | Piscivore | 1.00 | NonComm | -3.16 | 18378.87 | High | -7.45 | Plaice |
| Pollock | POLLACHIUS VIRENS | Piscivore | 5142.51 | Medium | -2.41 | 1.00 | NonComm | -11.65 | Pollock |
| Radiated Shanny | ULVARIA SUBBIFURCATA | Sm.Benthivore | 1.00 | NonComm | NA | 1.00 | NonComm | NA | NA |
| Red_Crab | GERYON QUINQUEDENS | Decapod.Benthivore | 1.00 | NonComm | NA | 1.00 | NonComm | -12.50 | Red_Crab |
| Red_Hake | UROPHYCIS CHUSS | Zoopiscivore | 1.00 | NonComm | 4.34 | 9192.20 | High | 6.63 | Red_Hake |
| Redfish | SEBASTES FASCIATUS | Zoopiscivore | 8578.21 | Medium | 0.02 | 3174.64 | Medium | -0.10 | Redfish |
| Rock_Crab | CANCER IRRORATUS | Decapod.Benthivore | 1.00 | NonComm | 0.00 | 83681.38 | High | -0.31 | Rock_Crb |
| Sandlance | AMMODYTES DUBIUS | Planktivore | 1.00 | NonComm | -19.75 | 4.10 | Low | -34.11 | Sandlanc |
| Scallop | PLACOPECTEN MAGELLANICUS | FilterFeeder | 84578.93 | High | 0.00 | 26159.27 | High | 1.61 | Scallop |
| Sea Raven | HEMITRIPTERUS AMERICANUS | Piscivore | 1.00 | NonComm | -5.43 | 12.70 | Low | -5.69 | SeaRaven |
| Shortfin_Squid | ILLEX ILLECEBROSUS | Piscivore | 1.04 | Low | 21.73 | 3489.42 | Medium | 12.96 | Shrtfn_S |
| Silver_Hake | MERLUCCIUS BILINEARIS | Zoopiscivore | 4390.96 | Medium | 6.00 | 7041.81 | Medium | 4.95 | Silvr_Hk |
| Smooth_Skate | MALACORAJA SENTA | Md.Benthivore | 1.00 | NonComm | -5.07 | 1.00 | NonComm | -12.92 | Smth_Skt |
| Snow_Crab | CHIONOECETES OPILIO | Decapod.Benthivore | 88949.46 | High | -6.18 | 1.00 | NonComm | 0.00 | Snow_Crb |
| Summer_Flounder | PARALICHTHYS DENTATUS | Md.Benthivore | 1.00 | NonComm | NA | 9644.88 | High | 12.90 | Smmr_Fln |
| Thorny_Skate | AMBLYRAJA RADIATA | Piscivore | 1.00 | NonComm | -2.47 | 1.00 | NonComm | -4.21 | Thrny_Sk |
| Turbot | REINHARDTIUS HIPPOGLOSSOIDES | Piscivore | 64.36 | Low | -5.90 | 1.00 | NonComm | NA | Turbot |
| White_Hake | UROPHYCIS TENUIS | Piscivore | 4390.96 | Medium | 3.56 | 1750.31 | Medium | 9.65 | White_Hk |
| Windowpane | SCOPHTHALMUS AQUOSUS | Md.Benthivore | 1.00 | NonComm | -11.54 | 159.29 | Low | -4.50 | Windowpn |
| Winter_Flounder | PSEUDOPLEURONECTES AMERICANUS | Md.Benthivore | 963.25 | Medium | -0.61 | 3848.62 | Medium | -5.29 | Wntr_Fln |
| Winter_Skate | LEUCORAJA OCELLATA | Lg.Benthivore | 1.00 | NonComm | -9.58 | 1.00 | NonComm | -3.87 | Wntr_Skt |
| Witch_Flounder | GLYPTOCEPHALUS CYNOGLOSSUS | Md.Benthivore | 963.25 | Medium | 1.31 | 4794.73 | Medium | -3.53 | Wtch_Fln |
| Wolffish | ANARHICHAS LUPUS | Lg.Benthivore | 1.00 | NonComm | -4.94 | 1.00 | NonComm | NA | Wolffish |
| Yellowtail_Flounder | LIMANDA FERRUGINEA | Md.Benthivore | 963.25 | Medium | -0.90 | 7698.66 | Medium | -5.76 | Yllwtl_F |
