# Supplementary material for: Thermal Habitat Index of Many Northwest Atlantic Temperate Species Stays Neutral under Warming Projected for 2030 but Changes Radically by 2060
Source: PLoS One. 2014 Mar 5;9(3):e90662. doi: 10.1371/journal.pone.0090662 (PMC3944076; doi:10.1371/journal.pone.0090662)
Supplement: File S1 — Supplementary Information on Surveys and Analyses. (DOCX) [file pone.0090662.s004.docx]

**File S1. Supplementary Information on Surveys and Analyses**

*Scientific trawl surveys*

The data used in our analyses came from scientific trawl surveys conducted by Fisheries and Oceans Canada on the Scotian Shelf, Bay of Fundy and Georges Bank, and by the National Marine Fisheries Services of the National Oceanic and Atmospheric Administration on the Northeast continental shelf of the United States, on Georges Bank,the Gulf of Maine, Southern New England and mid-Atlantic Bight. Table S1 shows an example of the bottom temperature summarized for the cod (*Gadus morhua)* dataset. The detailed survey methods are available in Doubleday ([1]) and are summarised here. The basic sampling unit of the survey consists in a 30-minute trawl tow at a speed of 3.5 knots. Under ideal conditions this procedure samples a length of 1.75 nautical miles and the swept area depends on the type of trawl gear used. However, for our purpose, the presence or absence of a given species is comparable between tows and the effect of different sampling gears can be ignored.

*Generalized additive models*

We used generalized additive model (GAM) to analyse the presence/absence data obtained from the scientific trawl survey data from Canada and the United States. Since a number of environmental covariates are recorded with each survey tow we were able to model the probability of occurrence of each species based on key parameters explaining their distribution. Generalized additive models were chosen for their ability to predict the binomial variable of interest, the presence/absence of each species in each sample. The distinction between GAMs and other modelling approaches is in the way that error terms are treated and for the inclusion of smoothing terms ([2]). Most other modelling approaches (e.g. generalized linear models) assume multiplicative error terms which can be a hindrance when dealing with explanatory variables whose parametric relationships are unknown. The main drawback from GAMs relates to the difficulty in the interpretability of its parameter estimates.

The GAM we used for the analyses presented had a binomial error structure that was properly suited for the presence/absence data used and included important environmental covariates. When appropriate, the covariates were also defined along intervals of potential values (so-called “knots” in GAM terminology) so as to properly estimate the probability of occurrence of species based on the explanatory variables Knots for temperature and depth were specified as 3 and 4, respectively, in The exact model we used for each species $i$ was:

$$logit\left( p\left( presence \right) \right)=\alpha+year+bottom T+depth+\left( longitude,latitude \right)$$

We acknowledged that our net change in realized habitat estimate was dependent on probability, which is dependent on sample size. To ensure that the fitted models were robust in predicting net change, we fit the model to the full data, and then subsampled the data, 10 times, in order to establish the accuracy and bias of realized habitat estimate.

References

1. Doubleday W (1981) Manual on groundfish surveys in the Northwest Atlantic. NAFO Sci Counc Stud 2: 56pp.

2. Wood S (2006) Generalized Additive Models: An Introduction. Boca Raton, Florida.: R. Chapman and Hall/CRC.

3. Shackell NL, Bundy A, Nye JA, Link JS (2012) Common large-scale responses to climate and fishing across Northwest Atlantic ecosystems. ICES J Mar Sci 69: 151–162. doi:10.1093/icesjms/fsr195.
